# The Crystallinity Control of Polymer Donor Materials for High-Performance Organic Solar Cells

**DOI:** 10.3389/fchem.2020.603134

**Published:** 2020-11-24

**Authors:** Dingding Qiu, Muhammad Abdullah Adil, Kun Lu, Zhixiang Wei

**Affiliations:** ^1^Chinese Academy of Sciences Key Laboratory of Nanosystem and Hierarchical Fabrication, Chinese Academy of Sciences Center for Excellence in Nanoscience, National Center for Nanoscience and Technology, Beijing, China; ^2^University of Chinese Academy of Sciences, Chinese Academy of Sciences, Beijing, China

**Keywords:** polymer solar cell, crystallinity, donor, aggregation, bulk hetereojunction

## Abstract

Bulk heterojunction (BHJ) organic solar cells (OSCs) can be regarded as one of the most promising energy generation technologies for large-scale applications. Despite their several well-known drawbacks, the devices where polymers are employed as the donor are still leading the OSC universe in terms of performance. Such performance generally depends upon various critical factors such as the crystallinity of the material, the crystallization process during the film formation, and also the final film morphology. Despite a few reviews on the structure of the polymer donor materials and device performance, not enough attention has been paid toward the crystallinity problem. Herein, the structure and crystallinity of the representative polymer donor materials and the corresponding device properties have been briefly reviewed. Furthermore, several typical methods for controlling the crystallinity of materials have been summarized and illustrated as well. Moreover, the obstacles lying in the way of successful commercialization of such polymer solar cells have been systematically discussed. The in-depth interpretation of the crystallinity of the polymer donors in this article may stimulate novel ideas in material design and device fabrication.

## Introduction

Bulk heterojunction (BHJ) organic solar cells (OSCs) have recently achieved extremely high power conversion efficiencies (PCEs) exceeding 18% owing to their interesting trademark properties (Liu et al., 2020). Over the past several decades, the researchers have put in tremendous efforts in the development and improvement of the corresponding materials (Li et al., 2005; Liao et al., 2013; Zhang et al., 2015), exploring alternate and better preparation methodologies (Gurney et al., 2019; Hu et al., 2020; Wang et al., 2020), presenting elaborate and accurate mechanisms for proving their findings, and so on (Ji et al., 2020; Meng et al., 2020). Hence, the parameters defining the OCS performance; the open circuit voltage (*V*_OC_), short circuit current (*J*_SC_), and the fill factor (FF), have been on a continual rise, which is a welcoming characteristic to meet the requirements of industrial applications (Cui et al., 2019; Liu et al., 2020).

**Figure 1:**
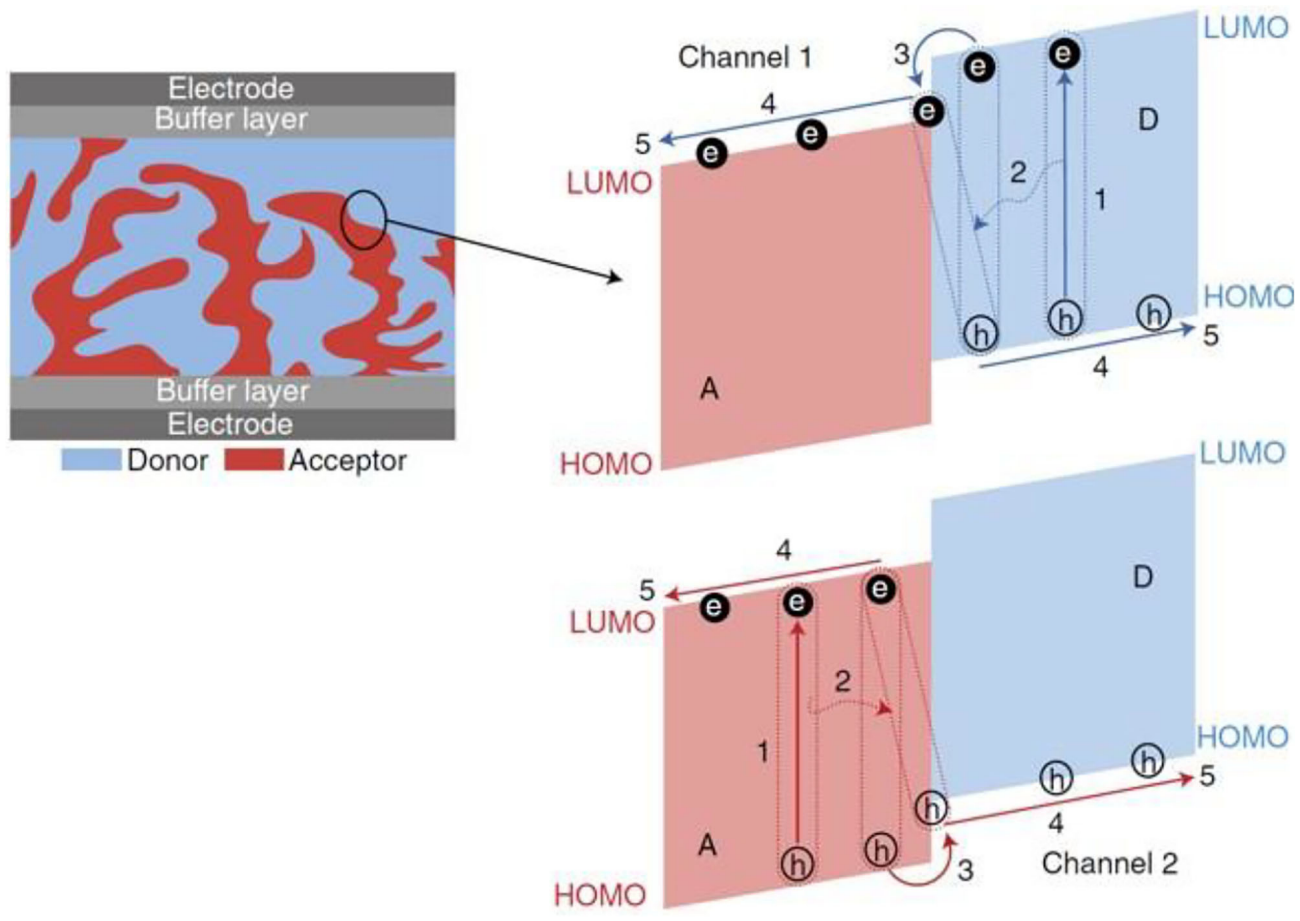
The operating mechanism of OPVs: (1) absorption of photons and creation of excitons; (2) diffusion of excitons to D/A interfaces; (3) dissociation of excitons to free charge carriers (holes and electrons) at D/A interfaces; (4) transportation of the charge carriers to electrodes; (5) extraction of the charge carriers at the electrodes. Copyright 2018, Macmillan Publishers Limited, part of Springer Nature.

The widely accepted working mechanism of the BHJ OSCs is illustrated in Figure 1. First, the donor and acceptor materials absorb the incident photons to generate excitons, which are basically electron and hole pairs. Next, these excitons diffuse toward the donor–acceptor interface, where after overcoming the Coulomb interaction force between them, the exciton gets separated into independent electrons and hole moieties. Finally, these independent electrons and holes transport toward the cathode and anode, respectively, and are collected by the corresponding electrodes.

It is generally believed that the selection of the appropriate active layer materials is one of the most important factors that affect the device performance. During the past few decades, such materials can be said to have undergone three stages of evolution. The first-generation materials were basically a combination of the famous poly(3-hexylthiophene) (P3HT) donor with a fullerene acceptor, where the resulting devices were capable of achieving PCEs of about 5% (Li et al., 2005). Later on, after the development of D-A copolymer systems and forming corresponding blends with fullerene derivatives, a massive improvement in the performance was observed as PCEs of over 11% were obtained (Zhao J. et al., 2016). Finally, the emergence of non-fullerene acceptors (NFAs) enabled the researchers to effectively control the OSC parameters, and ultimately, blending them with the matched high-bandgap polymer donors pushed the PCE values beyond a remarkable 17% (Xue et al., 2017; Cui et al., 2019). It is quite an extraordinary observation that despite their numerous drawbacks, currently, almost all the BHJ OSCs devices that exhibit excellent performance include polymers as their donor component.

In preparation of the OSCs, the crystallinity-related aspects are of major concern. A better way of understanding this important issue is to divide it into two parts, i.e., the crystallinity of the involved conjugated molecules, and the relationship between the crystallinity and the film morphology on device performance.

### The Crystallinity of Conjugated Molecules

Crystallinity, as the name suggests, describes the proportion of the ordered (crystalline) regions due to conjugation within a certain polymer and is a very important physical quantity in polymer science. Its value varies from polymer to polymer and hence there exists a wide range. Even for the same polymer, a slight variation in the processing conditions can lead to different crystallinity values. In BHJ OSCs, both polymer and small molecules possess large conjugated regions and therefore induce crystallinity in the system (Jiang et al., 2020). The source of crystallinity usually determines its influencing factors upon a certain system. Molecules with large conjugated regions demonstrate an extraordinary self-stability due to their large π-electron delocalization. Hence, through the overlapping of intermolecular π-electron clouds, these molecules can further achieve lower energies and therefore improved overall stability. As a result, the molecules with large conjugated planes have a certain tendency to self-aggregate, which is the source of their crystallinity (Manzhos, 2020). Therefore, the larger the molecular conjugated plane, the stronger will be the flatness of the stable conformation, which will lead to a stronger aggregation effect, and ultimately, stronger crystallinity will be attained. Also, factors such as the aggregation of alkyl chains and the non-covalent interaction between molecules have a certain impact on the molecular crystallinity.

### The Relationship Between Crystallinity and Film Morphology on Device Performance

The most widely utilized solution-based spin coating method to prepare thin OSCs active layers is essentially a crystallization process of multiple molecules. In this process, the phase separation occurs and largely determines whether or not the resulting film will form a nanoscale interpenetrating network structure, which in turn affects the performance of the final device (Bin et al., 2020). The crystallinity of the donor and acceptor molecules can be regarded as an important driving force for obtaining suitable phase separation for both polymers and small molecules and therefore determines the film's morphology and affects the final device performance (Heeger, 2014). Neither too strong nor too weak crystallinity is generally conducive to the formation of preferred morphology. Attaining the contrary to what has been said usually leads to poor device configurations.

**Figure 2:**
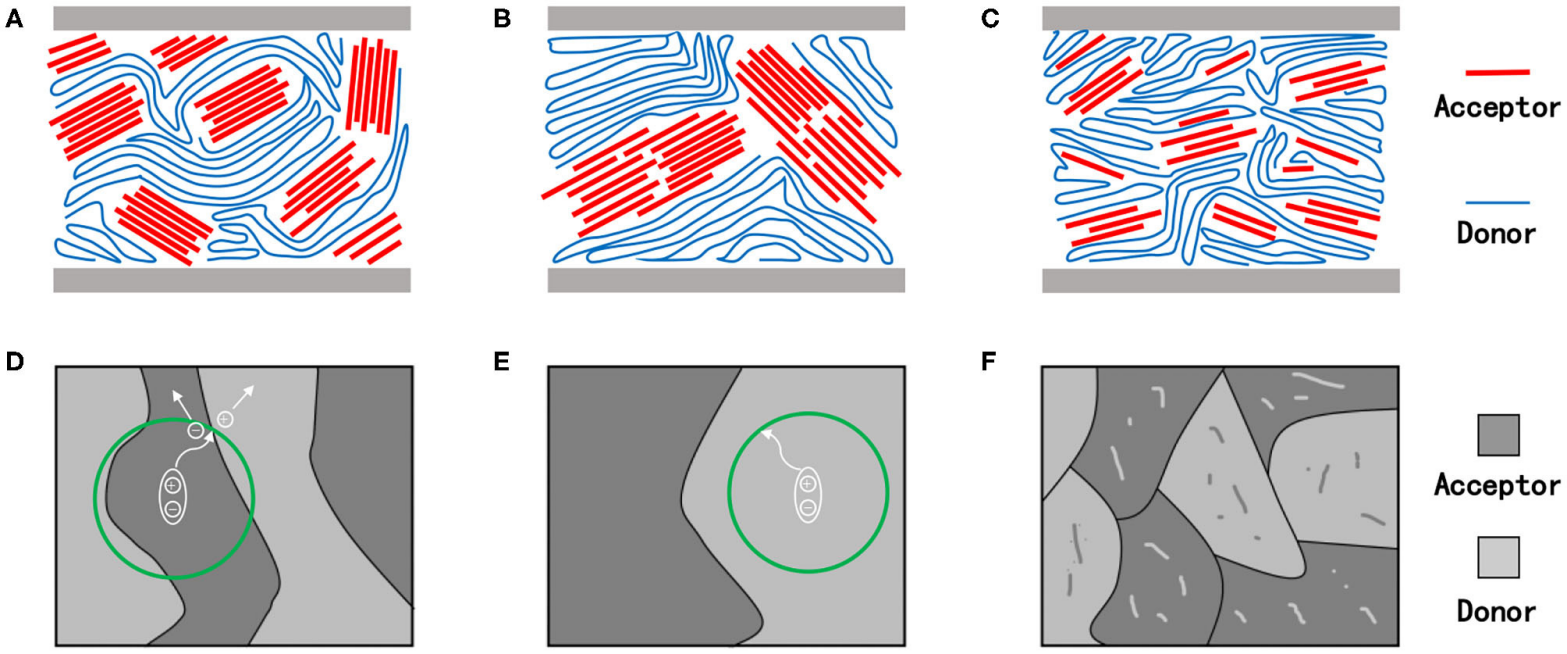
Schematic diagram of molecular stacking **(A–C)**, charge transfer and separation in the film **(D–F)** with suitable **(A,D)**, too strong **(B,E)**, and too weak **(C,F)** crystallinity. The green circle represents the range of electron-hole pairs transporting in organic semiconductor materials.

The desired molecular crystallinity, as well as the crystallization process for an ideal OSC, has been demonstrated in Figures 2A,D. Under the circumstance, the domain size of the active layer film should be equivalent to the exciton diffusion length of the involved materials while maintaining beneficial contact with the adjacent layers. Such a morphology, coupled with good phase purity, would enable the process of photoexcitation, exciton diffusion, charge separation, and charge transport in the device to be highly efficient, ultimately resulting in excellent device photoelectric performances (Bin et al., 2020). Therefore, attaining precise control of the crystallinity of materials and the crystallization process during device fabrication is important for obtaining high-performance devices.

Domains with excessive (too strong) crystallinity are illustrated in Figures 2B,E. As mentioned earlier, for a single molecule, the stronger its crystallinity, the greater will be its self-aggregation tendency, and the larger will be the domain size within the thin films, which will eventually lead to very high charge mobility (Guo et al., 2012). In blend films, however, the large domain size leads to modest phase separation and poor film morphology (Ge et al., 2020). Considering the short exciton lifetimes and about 10-nm diffusion length in organic semiconductor materials, the photogenerated excitons yielded in the active layer film with large domain size would not be able to reach the donor–acceptor interface to achieve proper charge separation, but will recombine and ultimately result in terrible devices performance (Heeger, 2014). In addition, the excessively large domain sizes are usually accompanied by relatively large film roughness and further lead to intimate contact with the hole and electron transport layers, resulting in heat generation and current loss from the corresponding cells (Williams et al., 2012).

Likewise, extremely poor molecular crystallinity is also undesirable in pursuit of fabricating high-performing devices (Figures 2C,F) as it usually leads to poor film formation, as well as weak aggregation in the system, and thus, obtaining films with good morphology and high phase purity becomes difficult. Hence, when materials with low crystallinity are employed, amorphous regions are often observed in the corresponding active layers thin films, which ultimately hinder the charge transport and sacrifice the photovoltaic performances of the device (Li et al., 2016a).

Herein, we review the molecular structure, crystallinity, and crystallization behavior of typical polymer donor materials in the blend films, along with the methods to control them. We will first classify the existing polymer donor materials into five categories according to their structure. Then, a variety of reported crystallinity modification methods would be analyzed, which are mainly divided into molecular structure and post-processing conditions. Finally, we will put forward a few key challenges and efforts to overcome those challenges for the polymer donor materials for OSCs. We hope and believe that this article's overall introduction to the crystallinity problem in polymer donor materials may provide some valuable inspiration for the further development of this field.

## Polymer Donors for Efficient OSCs

### Polythiophene Derivatives

**Figure 3:**
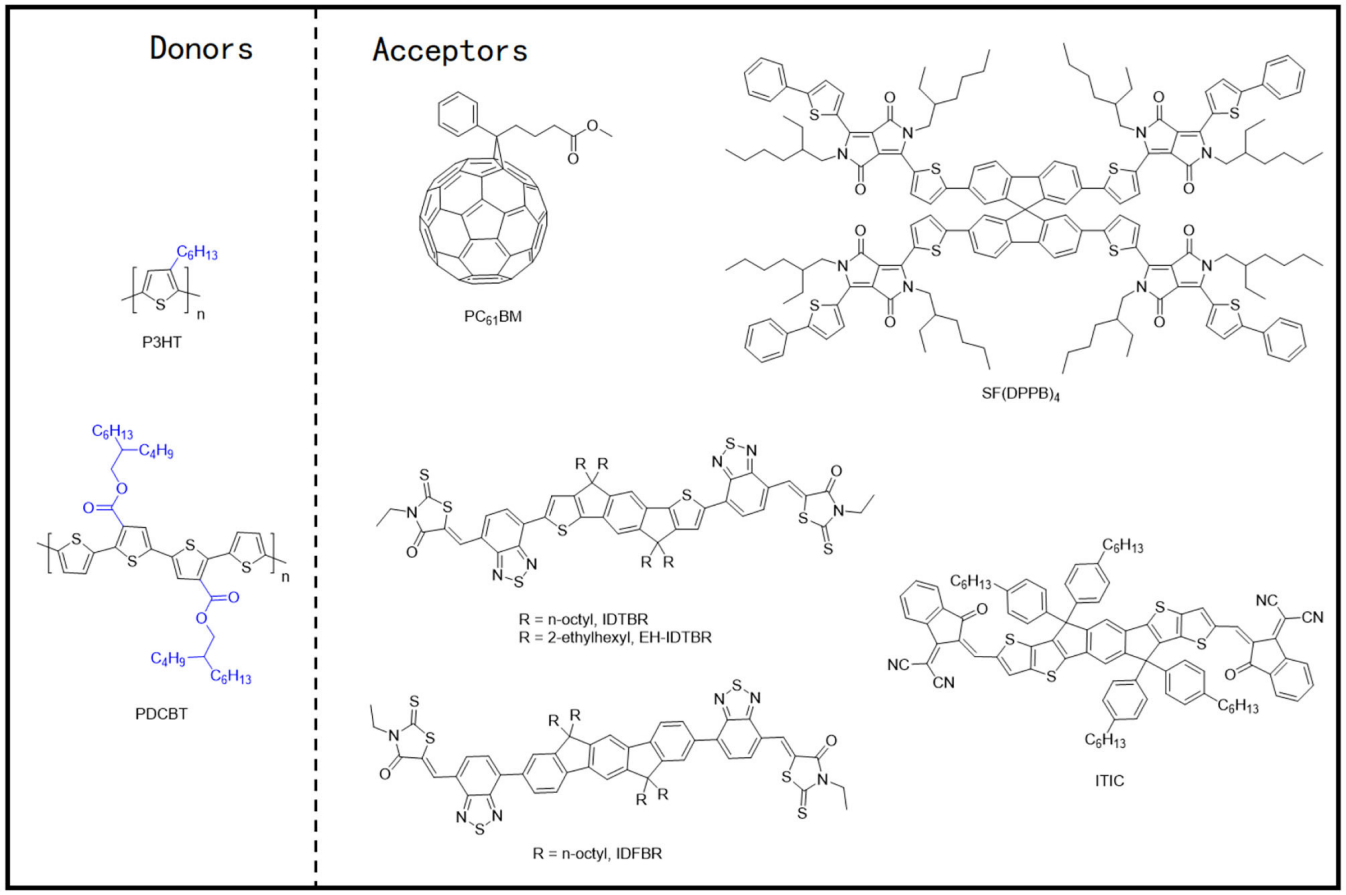
Molecular structures of the representative polythiophene derivatives and mentioned acceptors.

**Figure 4:**
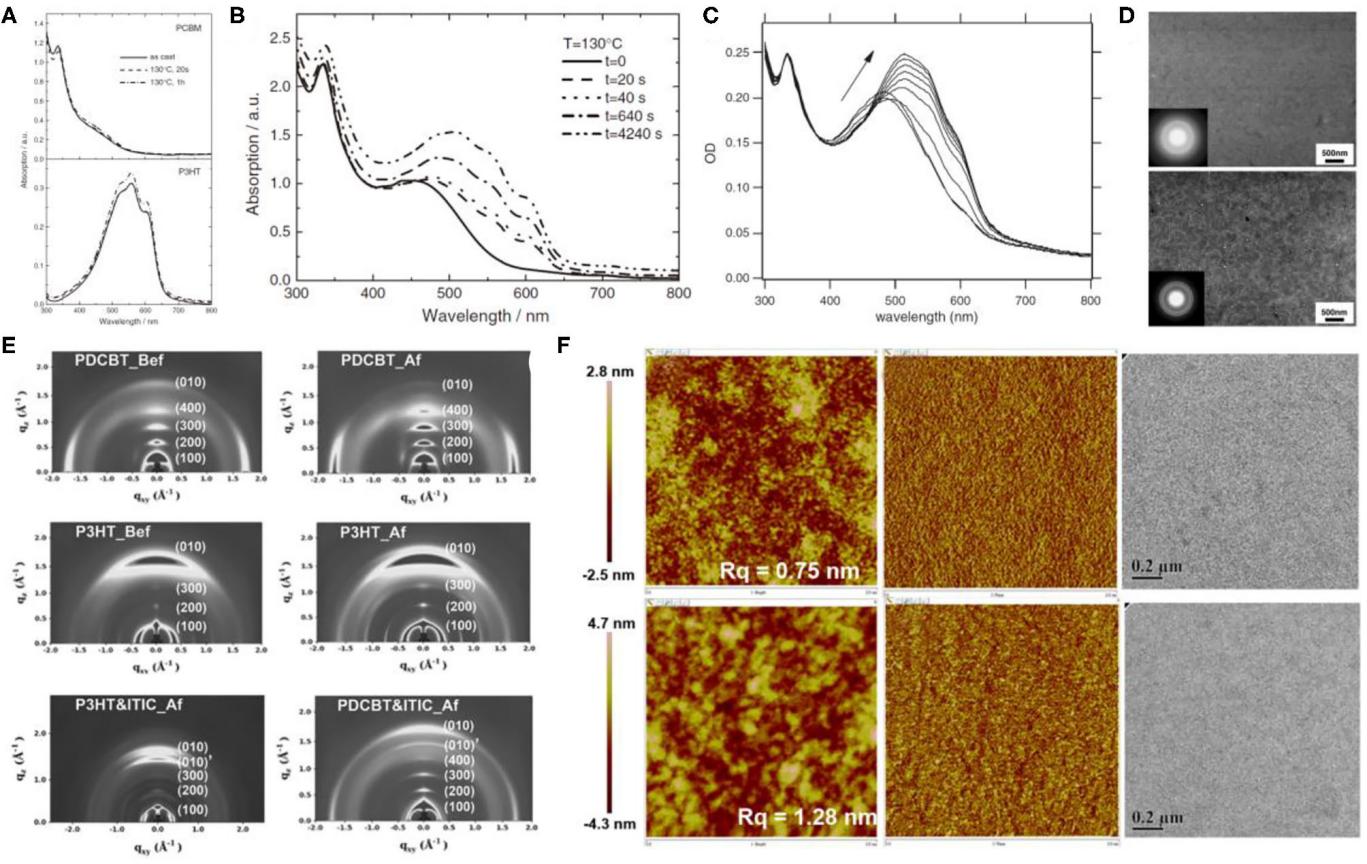
**(A)** Absorption spectra of pure PCBM (top) and P3HT (bottom) films as cast (solid curves), annealed at TA = 130°C for *t* = 20 s (dashed curves), and annealed for 1 h, TA = 130°C (dotted curves). Copyright 2004, IOP Publishing Ltd. **(B)** Absorption spectra of a P3HT:PCBM composite film as cast (solid curve) and after four successive thermal annealing steps, as indicated in the legend. The PCBM concentration is 67%. Copyright 2004, IOP Publishing Ltd. **(C)** OD spectra of PCBM:P3HT blend films directly after spin-coating and at 40, 50, 55, 60, 70, 80, 90, 100°C for 5 min. Arrow indicates rising temperatures. Copyright 2006, Elsevier B.V. **(D)** BF-TEM images of a 1:1 PCBM:P3HT pristine composite film (top) and after thermal annealing (bottom). The inserts are the corresponding selected area electron diffraction (SAED) patterns. Copyright 2006, Elsevier B.V. **(E)** 2D GIWAX images of the neat polymer and ITIC films. (Bef stands for before thermal annealing; Af stands for after thermal annealing.) Copyright 2016, Wiley-VCH. **(F)** Tapping mode AFM topography, phase, and TEM images of as-casted blend films based on the PDCBT:ITIC (top) and P3HT:ITIC (bottom). The sizes of the AFM images are 2 × 2 μm. The scale bars in the TEM images are 0.2 μm. Copyright 2016, Wiley-VCH.

During the early progression stages of the BHJ OSCs, polythiophene derivatives (PTs) used to be the most widely studied polymer donor materials as they were initially found to possess good photovoltaic characteristics. With the long polythiophene skeleton and alkyl side chains for solubilization, PTs hold both high crystallinity and good solution processability. P3HT (Figure 3) has been the most representative polythiophene-based polymer donor material in the field of OSCs. In one such example, Li et al. blended P3HT with [6,6]-phenyl-C61-butyric acid methyl ester (PC_61_BM) and achieved a PCE of 4.37% (Li et al., 2005). They suggested that the slower film formation rate brought higher molecular packing order in the blend, which can be indirectly proven by the increased film roughness, enhanced interband photon absorption, and improved mobility, and eventually led to an excellent photovoltaic performance. Later on, the crystallinity and morphology of the well-known P3HT:PCBM blends have been extensively studied. Comparing the absorption spectra of the pure P3HT and PCBM with their corresponding blend under various annealing times (Figures 4A,B) conclusively proves the crystallization of P3HT in the blend films (Chirvase et al., 2004). Similarly, the blend film exhibits a gradually increasing absorption peak at about 600 nm (Figure 4C) at increased annealing temperature, as well as a sharp signal peak (Figure 4E) in the GIWAXS spectrum, both of which reflect the strong crystallinity of the P3HT polymer (Savenije et al., 2006; Qin et al., 2016). The morphology of the PCBM:P3HT blend film has also been illustrated in Figure 4D, which exhibits a preferred nanoscale interpenetrating network structure. However, relatively lower *V*_OC_, *J*_SC_, and FF values have been attained as a consequence of utilizing a fullerene acceptor and an unoptimized film morphology.

Recently, the application of the NFAs has significantly improved the performance of the P3HT-based devices. In one such example, the indacenodithiophene (IDT) core–based NFAs, IDTBR, and EH-IDTBR, demonstrating highly planar molecular configuration, not only showed very good crystallinity, but also led to a highly complementary absorption spectrum with P3HT and hence produced an elevated PCE of 6.3 and 6%, respectively (Holliday et al., 2016). Interestingly, these two NFAs lost their face-on orientation after forming blends with the P3HT polymer, which might have reduced the devices' performance to a certain extent. Similarly, the performance of the P3HT based device was further improved by inserting a second NFA (IDFBR) into the P3HT:IDTBR blend film, forming a ternary system, which led to the added component induced optimization of phase morphology (Baran et al., 2017). On the contrary, introducing a spirobifluorene (SF) core–based NFA, SF(DPPB)_4_, with a cross-shaped molecular geometry, into the active layer with P3HT led to the generation of a remarkable *V*_OC_ of 1.41 V due to the energy level alignment. The corresponding devices ultimately produced a PCE of 5.16% (Li S. et al., 2016). From the results, it is evident that the face-on molecular orientation in the blend films has been achieved as the molecular geometry of SF(DPPB)_4_ prevented the strong aggregation of P3HT and strengthened the phase separation after thermal annealing.

It is widely accepted that the relatively large bandgap and high ionization potential of P3HT would cause weak light absorption and relatively low *V*_OC_ values that eventually undermine the device's performance. Therefore, great efforts have been inducted to ameliorate P3HT and develop new polythiophene donor materials. PDCBT, one of such outstanding polymer molecules (Figure 3), thus obtaining PCEs of 7.0% and 10.16% with PC_71_BM and ITIC as acceptors, respectively (Qin et al., 2016). The GIWAXS spectrum revealed that the alkoxycarbonyl-substituted PT showed enhanced crystallinity and preferred face-on molecular orientation (Figure 4E), which may be due to the dipole–dipole interactions between C=O (carbonyl)…S(thienyl) groups in PDCBT, and polar alkoxycarbonyl substituents and dicyanomethyleneindanone moieties in ITIC. As a result, a preferred film morphology and appropriate phase separation (Figure 4F) have been obtained and therefore led to improved device performance.

Despite the relatively inferior photovoltaic performances of PT-based devices, the advantages of low consumption and easy synthesis still make them one of the most promising donor materials for industrial applications. However, to do so, it is an urgent requirement to adjust the energy levels and absorption spectra of PT materials through simple side-chain engineering, halogenation strategies, or other easy methods.

### D-A Copolymer

With an alternating electron-rich unit (D) and an electron-deficient unit (A), the D-A copolymers are generally distinguished by their low bandgap, wide light absorption wavelength range, and adjustable characteristics of energy level and photon absorption. Because of the intramolecular push–pull electronic effects, simple side-chain engineering, fluorination strategies, etc., can affect the energy levels of the entire molecule and therefore change the optimal molecular configuration to modify the molecular stacking performance and crystallinity. Generally, the electron-deficient unit of the D-A copolymers is an ester substituted thieno[3,4-b]thiophene (TT), whereas the substituted benzo[1,2-b:4,5-b′]dithiophene (BDT) and 4,8-di(thiophen-2-yl)benzo[1,2-b:4,5-b′]dithiophene (BDT-T) are utilized as the electron-rich units.

#### D-A Copolymer Based on BDT Units

**Figure 5:**
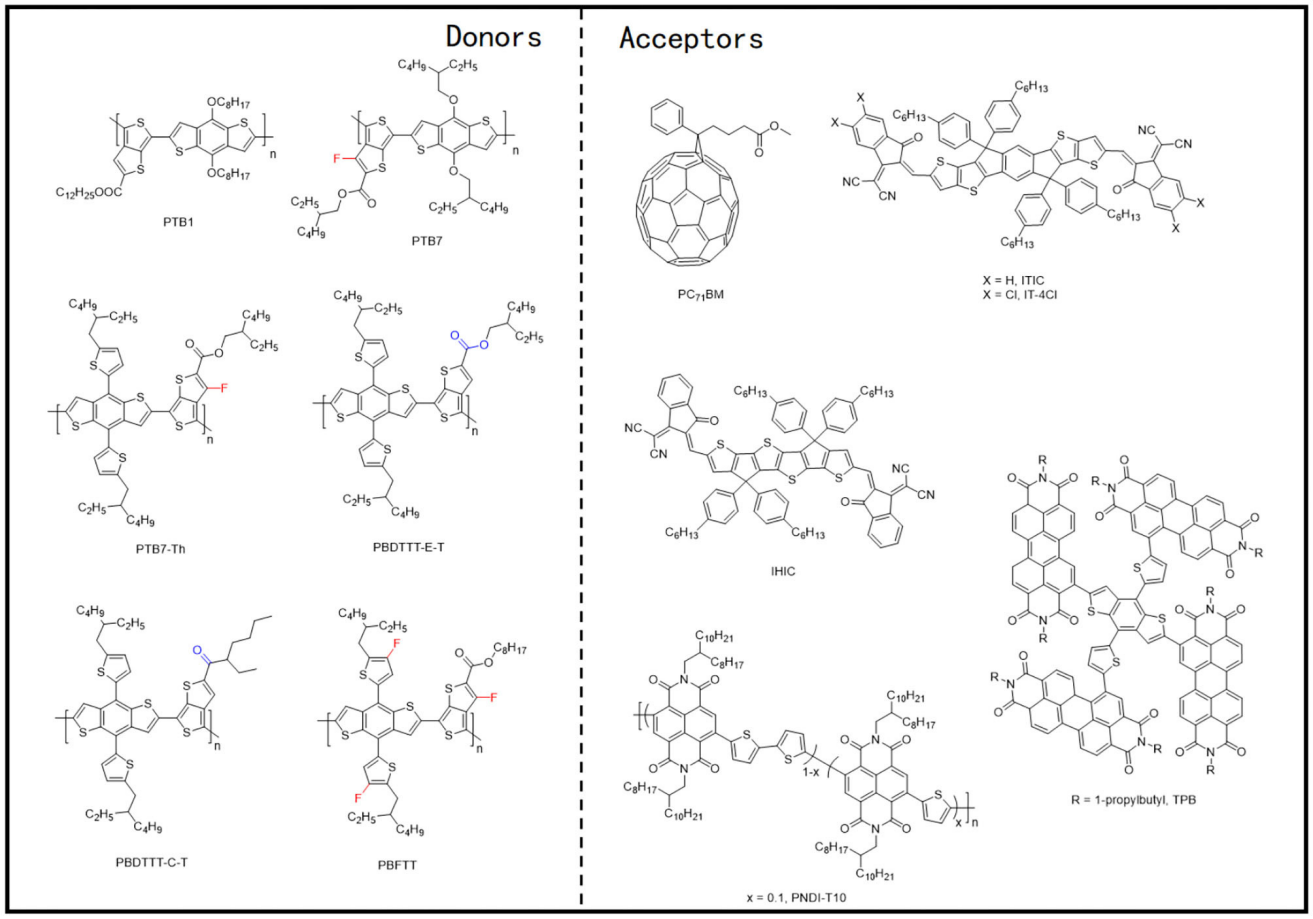
Molecular structures of representative D-A copolymers and mentioned acceptors.

**Figure 6:**
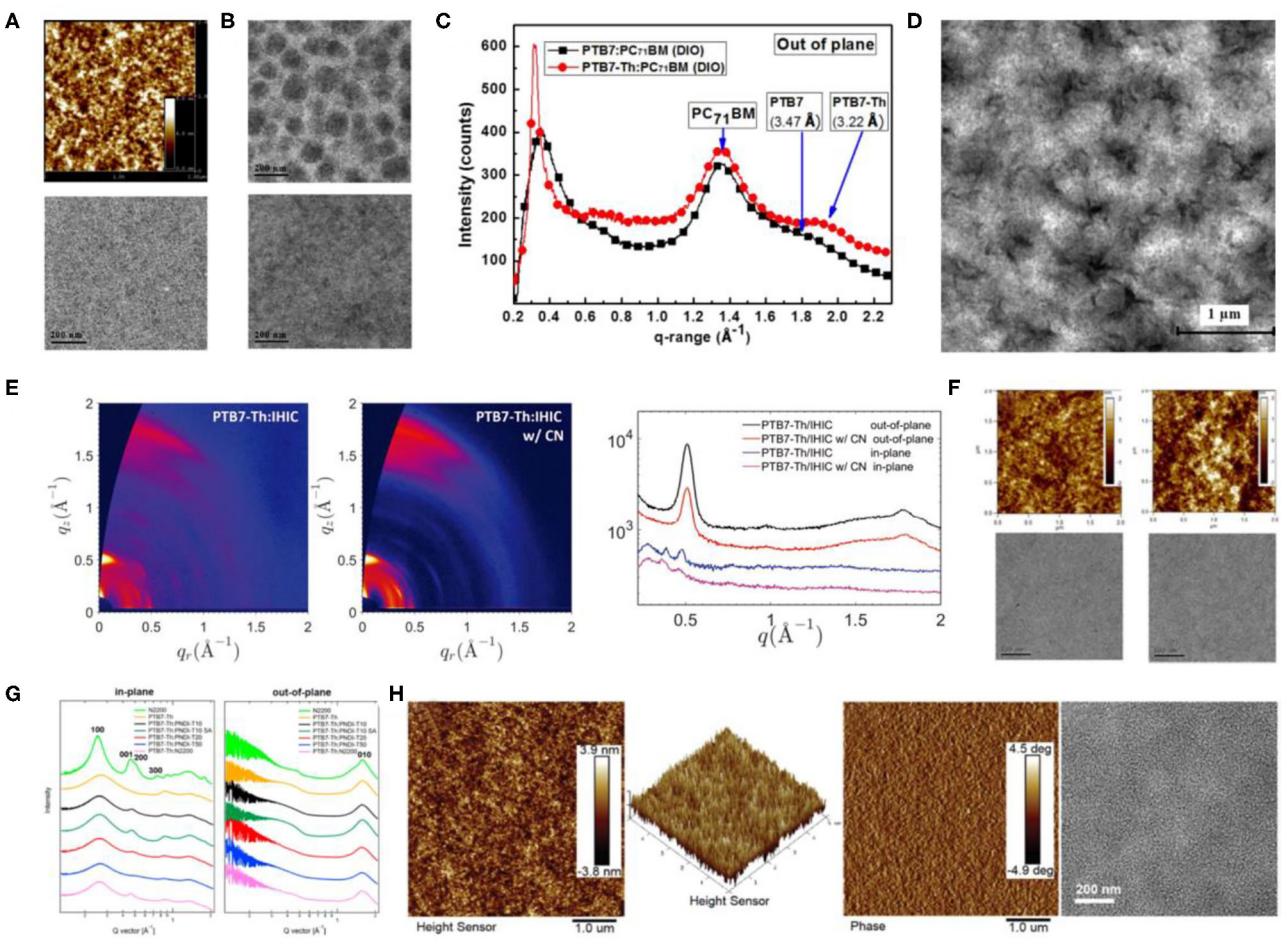
**(A)** AFM topography image (top) and TEM image (bottom) of PTB1/PC61BM blend films. Copyright 2009, American Chemical Society. **(B)** TEM images of PTB7/PC71BM blend film prepared from CB without (top) and with (bottom) DIO (the scale bar is 200 nm). Copyright 2010, Wiley-VCH. **(C)** Out-of-plane line cut of GIWAXS with PTB7:PC71BM and PTB7-Th:PC71BM (1:1.5 wt/wt, 100 nm). Copyright 2013, Wiley-VCH. **(D)** TEM image of PTB7-TH:ITIC (1:1.3, wt/wt) blended film. Copyright 2015, Wiley-VCH. **(E)** 2D GIWAXS patterns (left) of PTB7-Th:IHIC with and without 1-chloronaphthalene (CN) and the corresponding GIWAXS profiles (right) along the out-of-plane and in-plane directions. Copyright 2017, Wiley-VCH. **(F)** AFM (top) and TEM images (bottom) of TPB/PTB7-Th films (left) without additive and (right) 8% diphenyl ether (DPE) as additive. Copyright 2016, American Chemical Society. **(G)** In-plane and out-of-plane line cuts of GIWAXS patterns of PTB7-Th:PNDI-Tx and PTB7-Th:N2200 blend films. Copyright 2016, American Chemical Society. **(H)** AFM height image, 2D height image, phase image, and TEM images (from left to right) of optimal PBFTT:IT-4Cl blend films. Copyright 2019, Reproduced with permission.

In 2008, Liang et al. synthesized a polymer donor based on alternating TT and BDT units, called PTB1 (Figure 5), which achieved a PCE of 5.6% after being blended with PC_71_BM. They later extended the PBT series to PBT7 through side-chain and halogenation modification (Liang et al., 2009). Compared with P3HT, PTB1 displayed better molecular planarity and improved crystallinity, which upon blending with PC_71_BM brought a uniformly distributed nano-interpenetrating network structure [nanofibers structure of about ~5-nm width (Figure 6A)]. With attributes such as a narrow bandgap (1.6 eV) and high hole mobility (4.5 × 10^−4^ cm^2^/V · s), OSCs based on PTB1:PC_71_BM resulted in better device performance than the P3HT:PCBM system. After replacing the alkyl chain with 2-ethylhexyl and fluorinating the TT unit, PTB7 (Figure 5) was synthesized, and the PCE of the devices with the same acceptor material reached 7.4% (Liang et al., 2010). Elevated *V*_OC_, derived from fluorination, and 1,8-diiodooctane (DIO) doping–induced uniform phase separation (Figure 6B) favoring charge separation and transfer can be credited as the main reasons for the excellent performance of the PTB series donor–based devices.

#### D-A Copolymer Based on BDT-T Units

Later on, one of the most investigated polymer molecules, PTB7-Th, rose to the scene (Figure 5), which was designed and proposed by Liao et al. through the incorporation of 2-ethylhexyl-thienyl group into the BDT unit (BDT-T) in PTB7 (Liao et al., 2013). This inclusion led to an improved co-planarity of the molecular backbone that not only resulted in a 25-nm red shift in the absorption edge but also induced a better π-π stacking and preferred face-on molecular orientation (shorter π-π stacking distances of 3.22 Å; Figure 6C). As a result, the PTB7-Th: PC_71_BM-based device gave a high PCE of 7.64%. Such excellent device performance gained researchers' attention and hence NFAs started to be employed with PTB7-Th. Lin et al. designed and synthesized one of the most representative NFA, ITIC and upon blending it with PTB7-Th, yielded a PCE of 6.80% due to the broad absorption, balanced charge transport, good donor/acceptor miscibility and proper phase-separation (Figure 6D) (Lin et al., 2015). The same group later synthesized an improved NFA, IHIC, which exhibited strong NIR absorption but weak visible absorption, and prepared an efficient semitransparent—ST-OSC with a PCE of 9.77% (Wang et al., 2017). The GIWAXS characterization (Figure 6E) illustrates that the PTB7-Th:IHIC blend film demonstrated relatively stronger crystallinity and preferential face-on oriented domains, leading to high and balanced charge mobility and good donor–acceptor compatibility. Similarly, The α-substituted PDI derivatives, TPB with twisted 3D structure, has also been employed as acceptors with PTB7-Th and gave a high PCE of 8.47%, which was attributed to the extraordinarily high *J*_SC_ values (>18 mA · cm^−2^). This high current value has been attained as a consequence of the increased contact area between the active layer and interfacial electrode, due to the smooth film morphology brought about by the DPE doping (Figure 6F) (Wu et al., 2016). Furthermore, PTB7-Th has also been blended with a naphthodithiophene (NDI)–based polymer NFA, PNDI-T10, which led to a PCE of 7.6% (Li et al., 2016b). A preferred domain size of 10–20 nm, as proved by the resonant soft X-ray scattering characterization, predominant face-on orientation (Figure 6G), and a balanced μ_h_/μ_e_ ratio of 2, contributing to efficient charge separation and transfer, has been attributed to this performance. In general, PTB7-Th can be termed as one of the most promising polymer donor materials, because of its narrow bandgap, strong absorption, suitable crystallinity, and good compatibility with most acceptors.

Side-chain engineering and halogenation strategies have also been conducted for PTB7-Th and produced several excellent OSCs. Huo et al. replaced the alkyl side chain on the thiophene of the BDT-T unit with alkoxycarbonyl and alkylcarbonyl and synthesized PBDTTT-E-T and PBDTTT-C-T (Figure 5) (Huo et al., 2011). Comparing with the corresponding alkyl-substituted analogs, PBDTTT-E-T and PBDTTT-C-T exhibited better thermal stabilities, red-shifted absorption spectra, lower HOMO and LUMO energy levels, and significantly higher hole mobility and hence led to greatly improved photovoltaic properties. Moreover, Su et al. fluorinated both BDT-F and the TT units and synthesized PBFTT polymer (Figure 5), which after being blended with IT-4Cl obtained a PCE of 11.1% for ST-OSC (Su et al., 2019). The fluorination not only led to a higher extinction coefficient and stronger crystallinity in the system but also endowed the blend films with small RMS roughness, smooth surface morphology, and uniform bulk morphology (Figure 6H), which fit perfectly with the thin electrodes required for ST-OSCs.

### D–π-A Copolymer With Thiophene π-Bridge

In recent years, the donor–π-acceptor (D–π-A) principle has widely been applied in the polymer donor material design, where an electron-rich unit (D), a π-bridge (π, usually thiophene), and an electron-deficient unit (A) alternately constitute the backbone structure of the polymer. Comparing with the earlier mentioned D-A copolymer donors, these possess larger conjugated planes, stronger absorption, and hence better device performance. Among the rich variety of D–π-A copolymers, the BDT-T is the most commonly used D unit, whereas benzo[1,2-c:4,5-c′]dithiophene-4,8-dione (BDD) and 2H-benzo[d][1,2,3]triazole (BTz) are usually employed as the A units.

#### BDT-T and BDD Unit–Based D–π-A Copolymer With Thiophene as the π-Bridge

**Figure 7:**
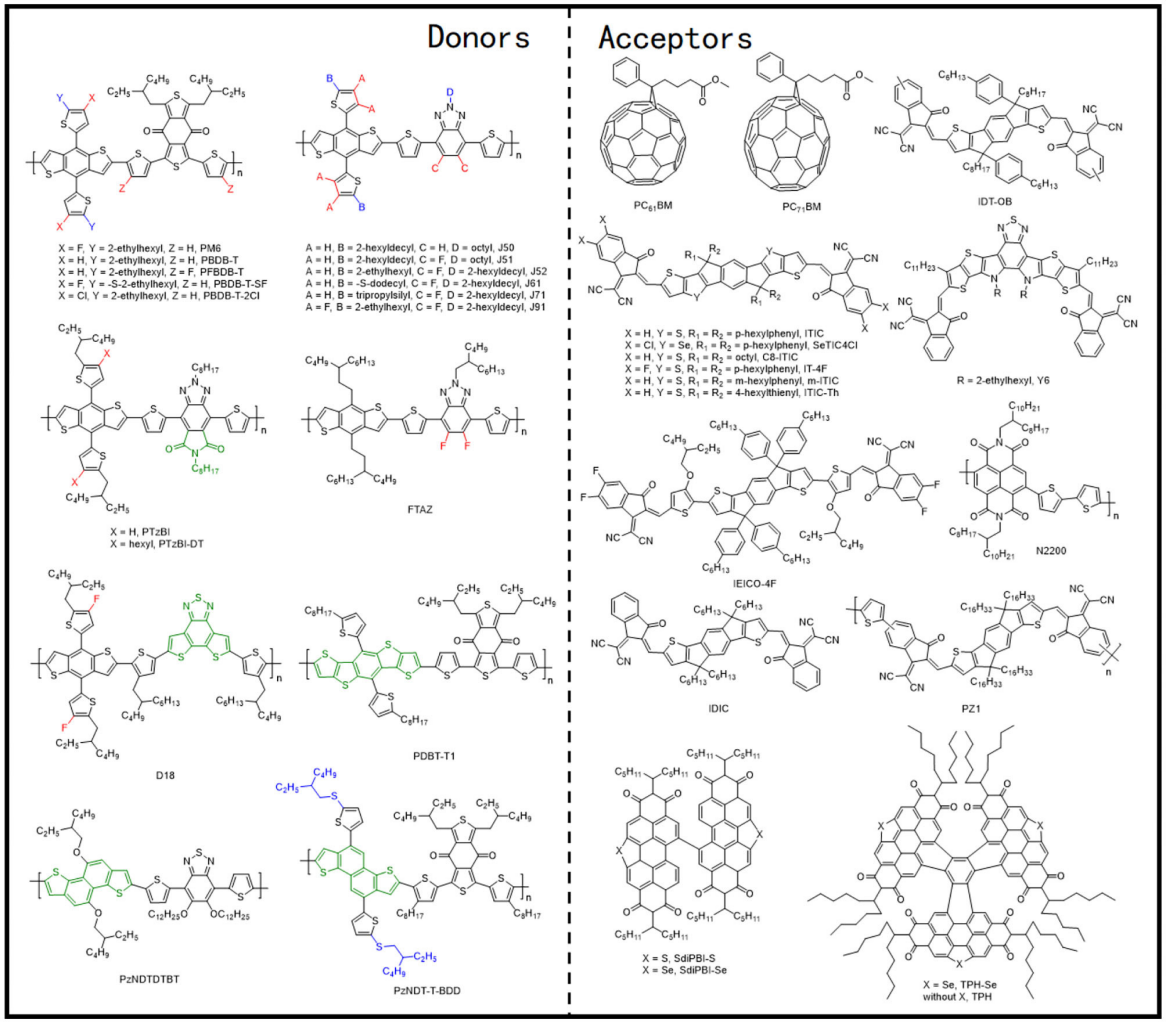
Molecular structures of representative D–π-A copolymers with T π-bridge and mentioned acceptors.

**Figure 8:**
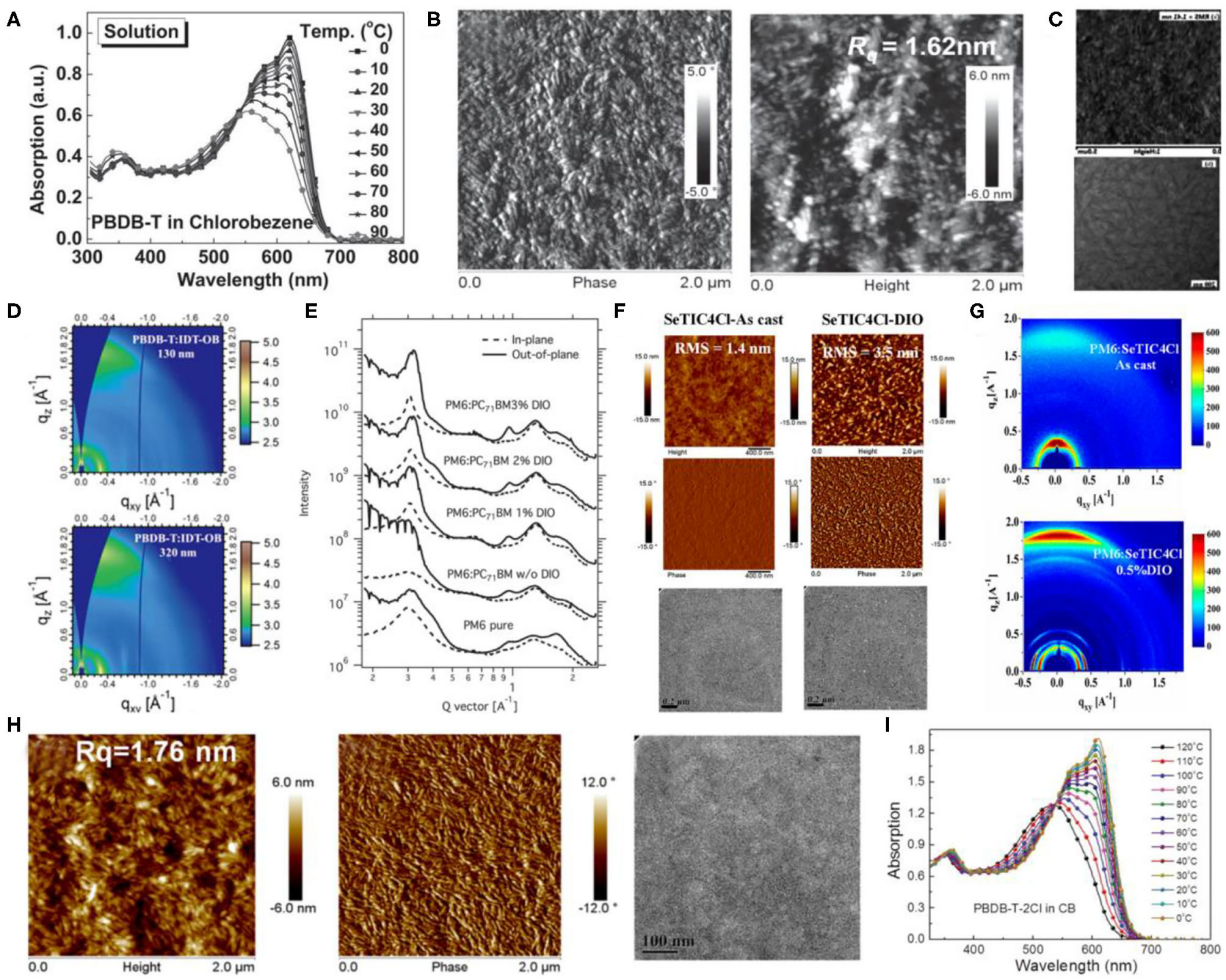
**(A)** Temperature-dependent absorption spectra of the polymer PBDB-T in chlorobenzene solution. Copyright 2016, Wiley-VCH. **(B)** AFM phase images (left) and AFM height images (right) of PBDB-T:ITIC film. Copyright 2016, Wiley-VCH. **(C)** AFM (top) and TEM (bottom) images of PBDB-T:IDT-OB film. Copyright 2017, Wiley-VCH. **(D)** 2D GIWAXS scattering patterns PBDB-T:IDT-OB blend films with different thickness of active layers. Copyright 2017, Wiley-VCH. **(E)** GIXD profiles for the pure film of PM6 and blend films of PM6:PC_71_BM (1:1.2, wt/wt) with different amounts of DIO. Copyright 2015, Wiley-VCH. **(F)** AFM height (top), phase (middle) and TEM (bottom) images of as-cast SeTIC4Cl/PM6 blend films (left) and DIO-treated SeTIC4Cl/PM6 blend films (right). Copyright 2018, American Chemical Society. **(G)** GIXD patterns of as-cast SeTIC4Cl/PM6 blend films and DIO-treated SeTIC4Cl/PM6 blend films. Copyright 2018, American Chemical Society. **(H)** Morphology images of the blend films: AFM height (left), phase (middle) images, and TEM image (right) of PBDB-T-SF:IT-4F blend film. Copyright 2017, American Chemical Society. **(I)** TD-Abs spectra of the solutions of PBDB-T-2Cl in CB as the temperature decreased from 120 to 0 °C. Copyright 2018, Wiley-VCH.

The PBDB-T polymer can be considered as the first widely studied polymer donor material based on the BDT-T and BDD units (Figure 7), which revealed a strong aggregation effect in the solution state (Figure 8A) and, when blended with the PC_61_BM as acceptor, exhibited a PCE of 6.67% (Qian et al., 2012). Likewise, upon utilizing the representative NFA, ITIC, with PBDB-T, the corresponding *J*_SC_ almost doubled and reached 16.81 mA · cm^−2^, ultimately producing an excellent PCE of 11.21%. Such a high performance was attributed to preferred film morphology and phase separation by the authors (Figure 8B) (Zhao W. et al., 2016). Furthermore, a fused-ring acceptor with asymmetric side chains, IDT-OB, has also been combined with PBDB-T, where the resultant devices were able to produce a PCE of 10.12%, as a consequence of suitable nanoscale phase separation (Figure 8C) and dominant face-on stacking orientation in a wide range of thickness (Figure 8D) (Feng et al., 2017). Moreover, constituting an all-polymer system, PZ1, a polymer NFA led to a relatively high PCE of 9.19% when blended with the PBDB-T donor (Zhang Z. G. et al., 2017).

Zhang et al. later on designed and synthesized PM6 (showed in Figure 7), another well-known copolymer of BDT-T and BDD units (Zhang et al., 2015). The PM6:PC_71_BM-based device showed a high PCE of 9.2% with a *V*_OC_ of 0.98 V, which is one of the highest fullerene acceptor–based device performances at that time. However, although the neat film of PM6 exhibited strong crystallinity and a dominant face-on packing with respect to the electrodes, the π-π stacking of the polymer in the blend films was completely disrupted after blending with PC_71_BM (Figure 8E), which is obviously unfavorable for charge transfer. The introduction of the NFAs solved this problem and greatly improved the photovoltaic performance of PM6-based devices. Later, the same group synthesized an NFA based on selenopheno[3,2-b]thiophene fused electron-rich central building block and end-capped electron-withdrawing group, named SeTIC4Cl (Wang et al., 2018), and ultimately, the corresponding PM6:SeTIC4Cl devices demonstrated a very high PCE of 13.32%. The AFM, TEM, and GIWAXS characterization revealed that the PM6 and SeTIC4Cl in the blend films can be crystallized independently, and the best face-on crystalline orientation and molecular packing can be obtained after DIO treatment (Figures 8F,G). Likewise, one of the most outstanding NFAs, BTP-4F, or more commonly known as the Y6 molecule, and its chlorinated homolog, Y6-Cl (BTP-4Cl), have been employed to constitute OSCs with PM6 as the donor and achieved record PCEs of 15.6 and 16.5%, respectively (Cui et al., 2019). Considering its popularity, Karki et al. examined the PM6:Y6 blend films to investigate the source of its excellent performance (Karki et al., 2019). They stated that the PM6:Y6 blend system exhibited a low energetic offset, a low energetic disorder, and beneficial morphology, which aids in reducing the voltage losses and retaining high FF and *J*_SC_ values simultaneously.

Hence, owing to the excellent photovoltaic performance of polymers based on BDT-T and BDD units, a lot of research has been conducted for their modification. In one such example, PFBDB-T (Figure 7) has been synthesized after fluorination on the π-bridge of PBDB-T. The corresponding PFBDB-T:C8-ITIC devices thus led to a high PCE of 13.2%, as a consequence of fluorination-dependent reduced energy losses within the blend (Fei et al., 2018). Similarly, Zhao et al. replaced the 2-ethylhexyl on the BDT-T unit of PM6 with a thioether group and obtained PBDB-T-SF polymer as shown in Figure 7. After blending it with IT-4F, the devices yielded a remarkable PCE of 13% (Zhao et al., 2017). Moreover, the PBDB-T-SF:IT-4F blend morphological properties were observed to be very similar to that of PBDB-T:ITIC blend films in terms of nanoscale phase separation and appropriate domain sizes (Figure 8H). In another example, PBDB-T-2Cl (Figure 7) has been successfully synthesized by substituting the fluorine atom on the BDT-T unit of the PM6 polymer with a chlorine atom, which displayed a temperature-dependent absorption spectrum in solution (Figure 8I). The PBDB-T-2Cl:IT-4F–based devices ultimately displayed a PCE of >14% with good stability (Zhang et al., 2018).

#### BDT-T and BTz Unit–Based D–π-A Copolymer With Thiophene π-Bridge

**Figure 9:**
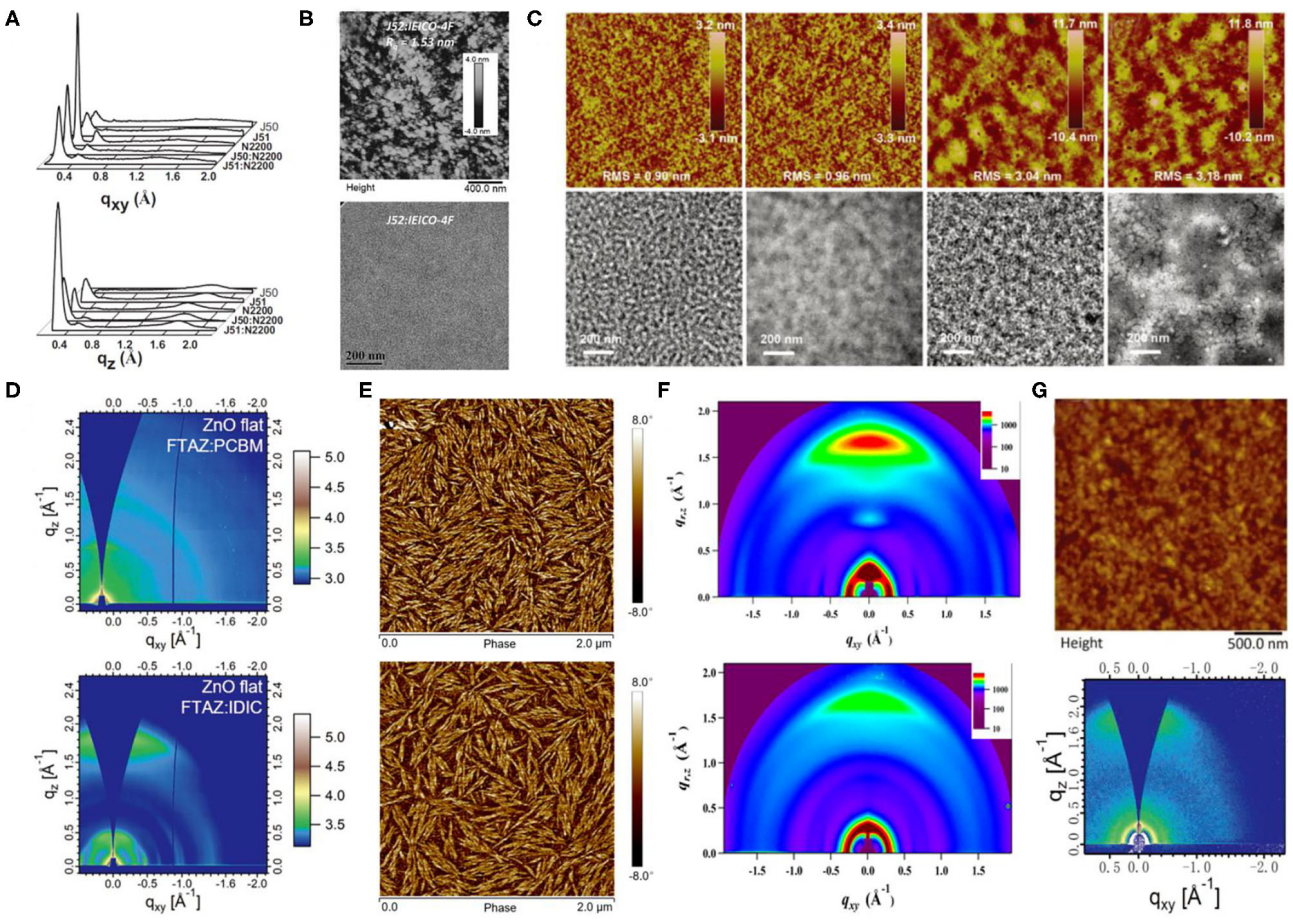
**(A)** Line cuts of GIWAXS images in in-plane (top) and out-of-plane (bottom) direction for the polymers J50, J51, N2200, and the polymer blends of J51:N2200 (2:1, wt/wt) and J50:N2200 (2:1, wt/wt) films. Copyright 2015, Wiley-VCH. **(B)** AFM height image (top) and TEM pattern (bottom) of the binary films at a donor ratio of 0.3:0.7. Copyright 2017, Wiley-VCH. **(C)** AFM height images (5 × 5 μm, top) and TEM images (bottom) for PTzBI:ITIC, PTzBI:ITIC processed with 0.5% dibenzyl ether (DBE), PTzBI-DT:ITIC and PTzBI-DT:ITIC processed with DBE/DIO (from left to right). Copyright 2017, Wiley-VCH. **(D)** The 2D GIWAXS patterns for FTAZ:IDIC and FTAZ:PCBM blended films with 0.25% DIO on flat ZnO substrates. Copyright 2018, Wiley-VCH. **(E)** AFM phase images for D18:Y6 blend films without (top) and with (bottom) SVA. Copyright 2020, Science China Press. **(F)** 2D GIWAXS patterns of (top) neat PDBT-T1 film and (bottom) optimal PDBT-T1:SdiPBI-Se blend film (0.25% DIO). Copyright 2015, American Chemical Society. **(G)** Tapping mode AFM topography (top) and 2D GIWAXS image (bottom) of PzNDT-T-BDD/IDIC blend film. The size of the AFM image is 2.5 × 2.5 μm. Copyright 2018, Wiley-VCH.

The BTz units generally have a hydrogen atom attached to the nitrogen atom in the system, which indicates the possibility of an additional alkyl chain attachment with the backbone that can enhance the solubilization. The J series polymer molecules have been the most reported polymers based on the BDT-T and BTz units. One of such polymers, J50 (Figure 7), has been synthesized via alternately connected alkyl-substituted BDT-T, thiophene π-bridge, and alkyl-substituted BTz units. Likewise, further modification of the acceptor unit utilizing the bifluorination strategy led to the synthesis of another impressive polymer, J51 (Figure 7) (Gao et al., 2016a). All polymer solar cells (PSCs) based on J51:N2200 demonstrated a high PCE of 8.27%, as a consequence of predominant face-on orientation (Figure 9A) and semicrystalline structure. Similarly, blending the J51 polymer with the ITIC acceptor led to a better complementary absorption spectrum and therefore further improved the efficiency to 9.26% (Gao et al., 2016b). Likewise, the J52 polymer has been synthesized by replacing the substituents on the donor and acceptor units with the most commonly used 2-ethylhexyl alkyl chain (Figure 7), and blending it with an ultranarrow bandgap NFA, IEICO-4F, led to a PCE of 9.3% with an excellent *J*_SC_ of 21.9 mA · cm^−2^ (Yao et al., 2017). The favorable nanoscale phase separation (Figure 9B), minor bimolecular recombination, and efficient overall charge collection processes have been attributed for such a performance from the corresponding combination. Later, further side-chain engineering, aimed at the BDT-T unit, has been carried out by substituting the alkyl chains by a thioether group, which resulted in the formation of J61 polymer. The corresponding device with *m*-ITIC acceptor resulted in a PCE of 11.77%, thanks to the better matched and fine-tuned donor–acceptor energy levels (Yang et al., 2016). Trialkylsilyl has also been employed as a substituent on the BDT-T unit to obtain a low HOMO copolymer J71 (Figure 7), whereby blending it with ITIC acceptor displayed a PCE of 11.41% (Bin et al., 2016). To take advantage of the fluorination effect, the same group difluorinated the BDT-T unit of J51 and obtained the J91 polymer (Figure 7). An impressive PCE of 11.63% was achieved after blending it with *m*-ITIC as a consequence of more intense absorption, low-lying HOMO energy level, and higher charge carrier mobility (Xue et al., 2017).

In addition to side-chain engineering and halogenation strategies, the BTz unit has also been modified by diimide functionalization, leading to the formation of novel acceptor materials, PTzBI and PTzBI−2-decyltetradecyl (DT) (Figure 7), which possessed improved crystallinity and preferred molecular packing orientation (Figure 9C), which endowed the corresponding devices with ITIC as acceptor with PCEs of 10.24 and 9.43%, respectively (Fan et al., 2017b).

#### Other D–π-A Copolymer With Thiophene π-Bridge

Similar to previous examples, the BDT unit has also been utilized to polymerize with the thiophene and BTz units, which led to the formation of another group of FTAZ (Figure 7) type of polymers. However, forming FTAZ: PC_61_BM blend films led to a system where both donor and acceptor exhibited weak scattering peaks, which indicated a weak molecular packing, and thus, the relevant OSCs showed a moderate PCE of 5.99% (Lin et al., 2018). Involving an NFA, IDIC, however, yielded an enhanced PCE of 12.14% as the corresponding devices benefited from improved crystallinity and dominant face-on orientation relative to the electrodes (Figure 9D). Another important D–π-A polymer that must be mentioned is D18 (Figure 7), which when blended with Y6 acceptor produced an outstanding efficiency of 18.22% (Liu et al., 2020). Such a remarkable performance has been attributed to the large molecular conjugate plane of BDT-T and DTBT units that promoted the polymer stacking and enhanced the μ_h_ value, as well as the evenly distributed nanofiber structure about 20 nm (Figure 9E). Likewise, combining another two thiophene rings on the base of BDT-T to extend the conjugated area, the DTBDT-T unit has also been introduced to the D–π-A polymer system, which led to the successful synthesis of several donor materials such as PDBT-T1 (Figure 7). Several perylene bisimide (PBI)–based small molecule acceptors with a twisted conformation have been blended with PDBT-T1, and hence, the OSC devices based on PDBT-T1:SdiPBI-S, PDBT-T1:SdiPBI-Se, PDBT-T1:TPH, and PDBT-T1:TPH-Se delivered PCEs of 7.16, 8.4, 8.28, and 9.28%, respectively (Sun et al., 2015; Meng et al., 2016a,b). These kinds of acceptors with strong aggregation effect showed good compatibility and preferred face-on orientation with PDBT-T1 in the blend films (Figure 9F). Thienyl side-chains functionalized ITIC-Th has also been mixed with PDBT-T1, and the corresponding device showed a PCE of 9.6%, which has been attributed to balanced charge transport and predominant intermolecular π-π interactions vertical to the substrates in the blend films (Lin et al., 2016).

Replacing the benzene of the BDT unit with naphthalene is also a common strategy to extend the conjugated area, where the obtained NDI is employed as an electron-rich unit in the D–π-A polymers. It is practically evident that the “zigzag” NDT (zNDT)–based D–A polymers often possess unique and excellent optoelectronic properties when combined with a suitable acceptor. Shi et al. combined NDI, benzo[c][1,2,5]thiadiazole (BT) unit, and thiophene π-bridge alternately and synthesized a polymer donor, named PzNDTDTBT (Figure 7) (Shi et al., 2013). Forming the corresponding blends with PC_71_BM showed a moderate PCE of 5.07% due to poor compatibility and large domain size. Later, copolymer PzNDT-T-BDD (Figure 7) consisting of zNDI and BDD units has been synthesized, and the resulting devices with IDIC as the acceptor showed an improved PCE of 9.72% (Jiang et al., 2018). Facile π-π stacking, appropriate absorption complementation, and active layer morphology have been credited for this performance enhancement (Figure 9G).

In general, the D–π-A copolymers are the most researched donor materials as they exhibit the best performances in the BHJ OSCs. Broad and strong absorption, strong crystallinity, good compatibility with NFAs, simple property controllability, and preferred face-on orientation in blend films grant them huge potentials for better device performance and even for commercial applications.

### Copolymers of Electron-Deficient Units and Bithiophene

**Figure 10:**
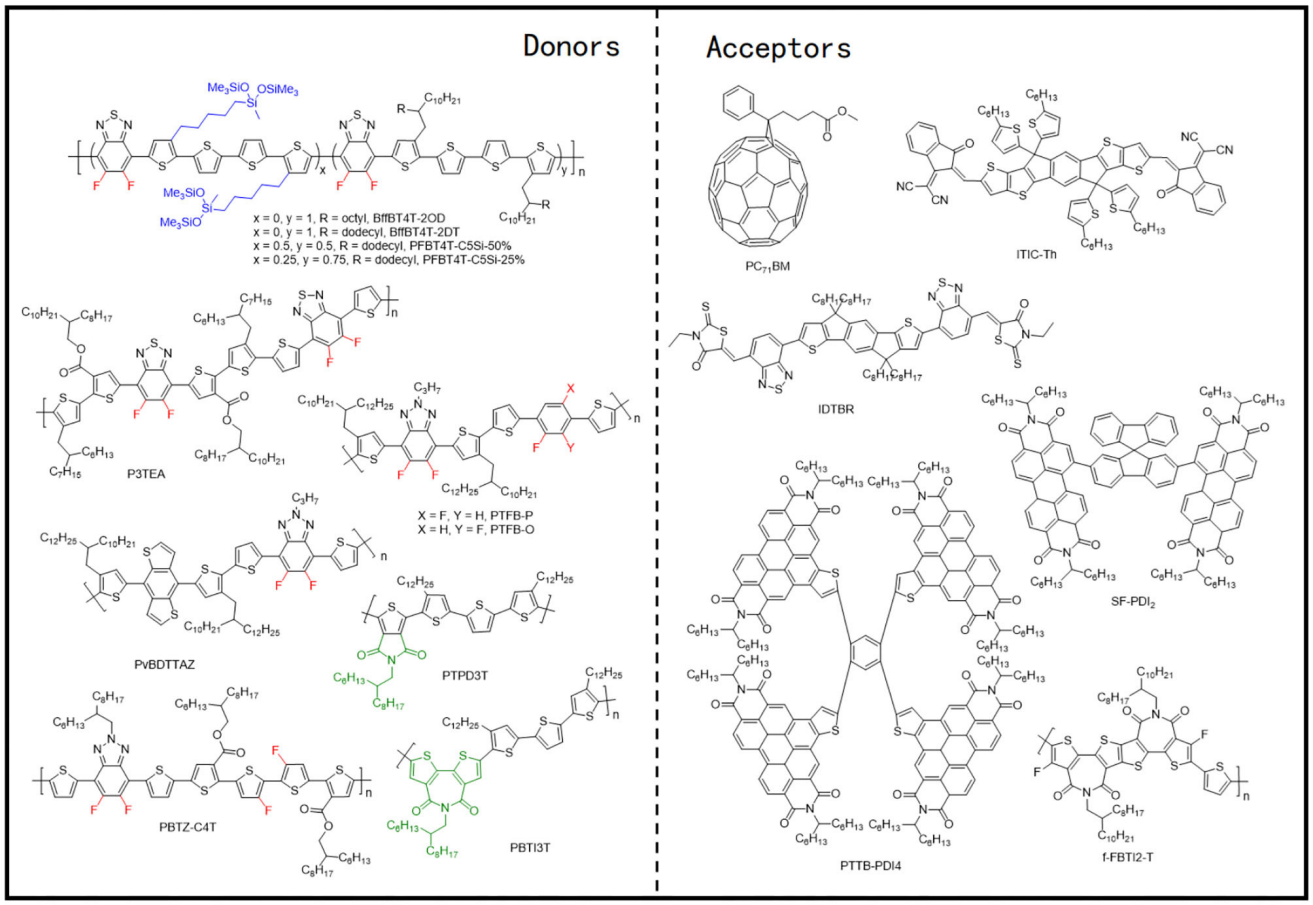
Molecular structures of representative copolymers of the electron-deficient unit and bithiophene and mentioned acceptors.

**Figure 11:**
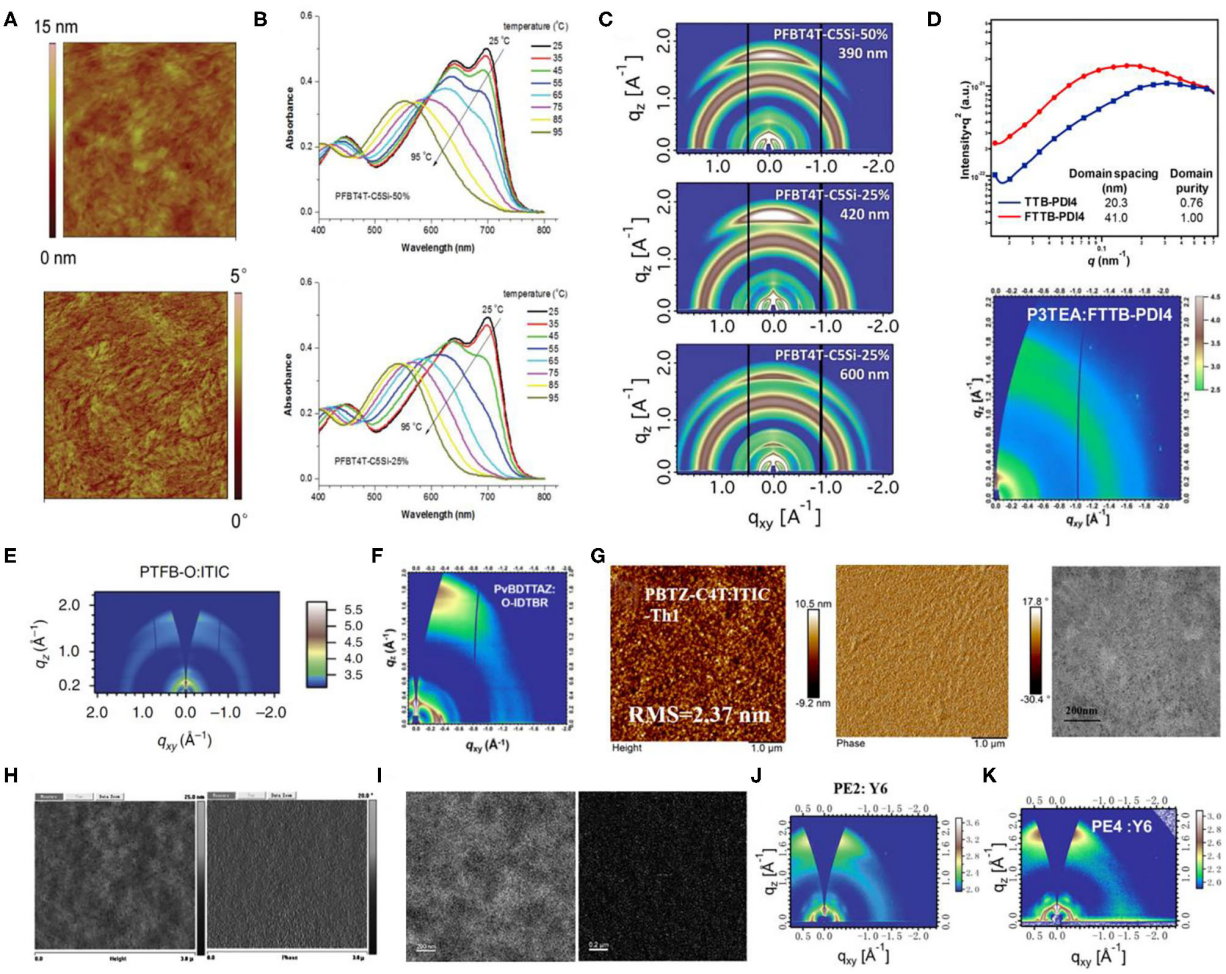
**(A)** AFM images (1 × 1 mm) of PffBT4T-2DT:SF-PDI2 blends. Copyright 2015, The Royal Society of Chemistry. **(B)** The evolutions of UV-vis absorption spectra of PFBT4T-C5Si-50% (top) and PFBT4TC5Si-25% (bottom) in chlorobenzene (1 × 10^−5^ M) during the heating process from 25 to 95°C. Copyright 2017, The Royal Society of Chemistry. **(C)** Grazing incidence X-ray diffraction (GIXD) 2D scattering images of thick BHJ films of PFBT4T-C5Si-50% and PFBT4T-C5Si-25%. Copyright 2017, The Royal Society of Chemistry. **(D)** RSoXS profiles (top) of P3TEA:TTB-PDI4 and P3TEA:FTTBPDI4 and 2D GIWAXS pattern (bottom) of P3TEA:TTB-PDI4. Copyright 2017, American Chemical Society. **(E)** Two-dimensional GIWAXS pattern of PTFB-O:ITIC blend film. Copyright 2016, Reproduced with permission. **(F)** 2D GIWAXS patterns for PvBDTTAZ:IDTBR films. Copyright 2017, American Chemical Society. **(G)** AFM and TEM images of PBTZ-C4T:ITIC-Th1 blend film. Copyright 2019, American Chemical Society. **(H)** AFM topography images (left) and phase-contrast images (right) of PBDTT–DTTBT/PC70BM (1:1.5) films. Copyright 2012, The Royal Society of Chemistry. **(I)** Zero-loss high-resolution phase-contrast TEM images (left) of PBDTT–ttTPD:PC71BM blends (1:1 wt/wt) cast from chloroform with 3 vol% DIO and nitrogen mapping ESI images (right) of PBDTT–ttTPD:PC71BM blends (1:1 wt/wt) cast from chloroform with 3 vol% DIO by a three-window method (N atom, 403.5 eV). Copyright 2014, The Royal Society of Chemistry. **(J)** The 2D GIWAXS patterns of the PE2:Y6 blend films. Copyright 2019, The Royal Society of Chemistry. **(K)** Two-dimensional GIWAXS (2D-GIWAXS) patterns of the PE4:Y6 blended films. Copyright 2019, American Chemical Society.

This system involves a relatively simple and operation-friendly methodology to take advantage of the modest synthesis techniques of the PTs, as well as the excellent performance of D-A copolymers simultaneously. The most common electron-deficient units used are BT and BTz. Besides, thieno[3,4-c]pyrrole-4,6-dione (TPD) and 2,2′-bithiophene-3,3′-dicarboximide (BTI) have also been reported. In this regard, the ffBT-based donor polymer, PffBT4T-2DT (Figure 10), has been synthesized by combining the BT and the tetrathiophene alternatively. Upon mixing it with an NFA with high-lying LUMO, SF-PDI_2_ led to a PCE of 6.3%, largely due to appropriate energy level matching and favorable BHJ morphology (Figure 11A) (Zhao et al., 2015). An unbalanced hole and electron mobility, however, resulted in unsatisfactory performance. Later, another NFA, IDTBR, has been employed with the same donor, and the corresponding devices displayed an improved PCE of 10%. This enhancement has been attributed to the low voltage losses and good luminescence yields in the blend films (Baran et al., 2016). Similarly, utilizing the side-chain engineering to replace the DT on the thiophene unit of PffBT4T-2DT with a 2-octyldodecyl, PffBT4T-2OT (Figure 10) has been synthesized. Hence, the OSCs based on PffBT4T-2OT:PC_71_BM achieved a PCE of 9.2%, thanks to an increase in both the domain size and exciton diffusion length during thermal annealing (Zhang et al., 2019). Furthermore, PFBT4T-C5Si-50% and PFBT4T-C5Si-25% have been synthesized by partly replacing the DT side chain using a siloxane-terminated side group (Figure 10), which demonstrated strong crystallinity and temperature-dependent solution absorption spectrum (Figure 11B) (Liu et al., 2017). Using PC_71_BM as an acceptor, the corresponding blend films exhibited preferred morphology and face-on stacking orientation even at film thicknesses up to 600 nm (Figure 11C). Similarly, P3TEA (Figure 10), another well-researched polymer whose structure comprises alternately connected BT unit and ester-substituted trithiophene, when blended with SF-PDI_2_, showed a PCE of 9.5% due to the efficient charge generation and separation, despite having a negligible driving force (Liu J. et al., 2016). Likewise, replacing the acceptor with a PDI-tetramer NFA, FTTB-PDI4, led to an improved PCE of 10.58% as a consequence of stronger π-π stacking and higher domain purity in the blend films (Figure 11D) (Zhang J. et al., 2017).

In another example, by inserting one BTz unit between every five thiophenes and performing ortho or para bifluorination on those thiophenes, Li et al. synthesized two polymer donors, PTFB-O and PTFB-P, as shown in Figure 10 (Li et al., 2016a). The corresponding devices based on PTFB-O:ITIC, due to suitable crystallinity (Figure 11E) and good donor and acceptor compatibility, showed a modest PCE of 9.9%. Chen et al. similarly added a BDT unit into the copolymer of BTz unit and bithiophene, which led to the synthesis of PvBDTTAZ (Figure 10). Mixing it with NFA IDTBR thus led to an impressive PCE of 11.6%, which was attributed to high crystallinity and small domains for both donor and acceptor within the blend films (Figure 11F) (Chen et al., 2017). Interestingly, ester substitution and fluorination have been applied to form a unique polymer donor, PBTZ-C4T (Figure 10), maintaining an A_1_-π-A_2_ architecture. Blending it with ITIC-Th led to a PCE of 9.34%, owing to a better planar geometry, improved light absorption, and high crystallinity (Figure 11G) (Guo et al., 2019).

Apart from the molecules mentioned above, TBD and BTI units have also been often employed to construct copolymer donor materials with bithiophene. Through copolymerization of TPD and BTI as the electron acceptor unit and terthiophene (3T) as the electron donor unit, Guo et al. synthesized PTPD3T and PBTI3T (Figure 10) and upon their incorporation with PC_71_BM acceptor, extremely high FFs of 76% to 80% with PCEs of about 8.7% have been achieved (Guo et al., 2013). The distinguished FF values have been attributed to close π-π interplanar spacings, face-on molecular oriented microstructures, ordered BHJ bicontinuous networks, and vertical phase gradation. Later, Sun et al. fused two fluorinated BTI units and combined it with a simple thiophene, obtaining a novel BTI-based polymer, f-FBTI2-T (Figure 10) (Sun et al., 2019). Because of the extended π-conjugation, reduced bandgap, and lower-lying LUMO energy level, the all-PSCs based on PTB7-Th:f-FBTI2-T exhibited an extraordinary PCE of 8.1%.

### D–π-A Copolymer With TT π-Bridge

TT units possess a rigid and coplanar fused ring, which ensures a highly delocalized π-electron system and strong intermolecular π-π stacking, and thus has attracted much interest for the construction of high performance polymer materials for applications in optoelectronic devices. Utilizing the TT as the π-bridge in D–π-A copolymer is a commonly used strategy to extend the conjugated plane and improve the crystallinity of materials.

**Figure 12:**
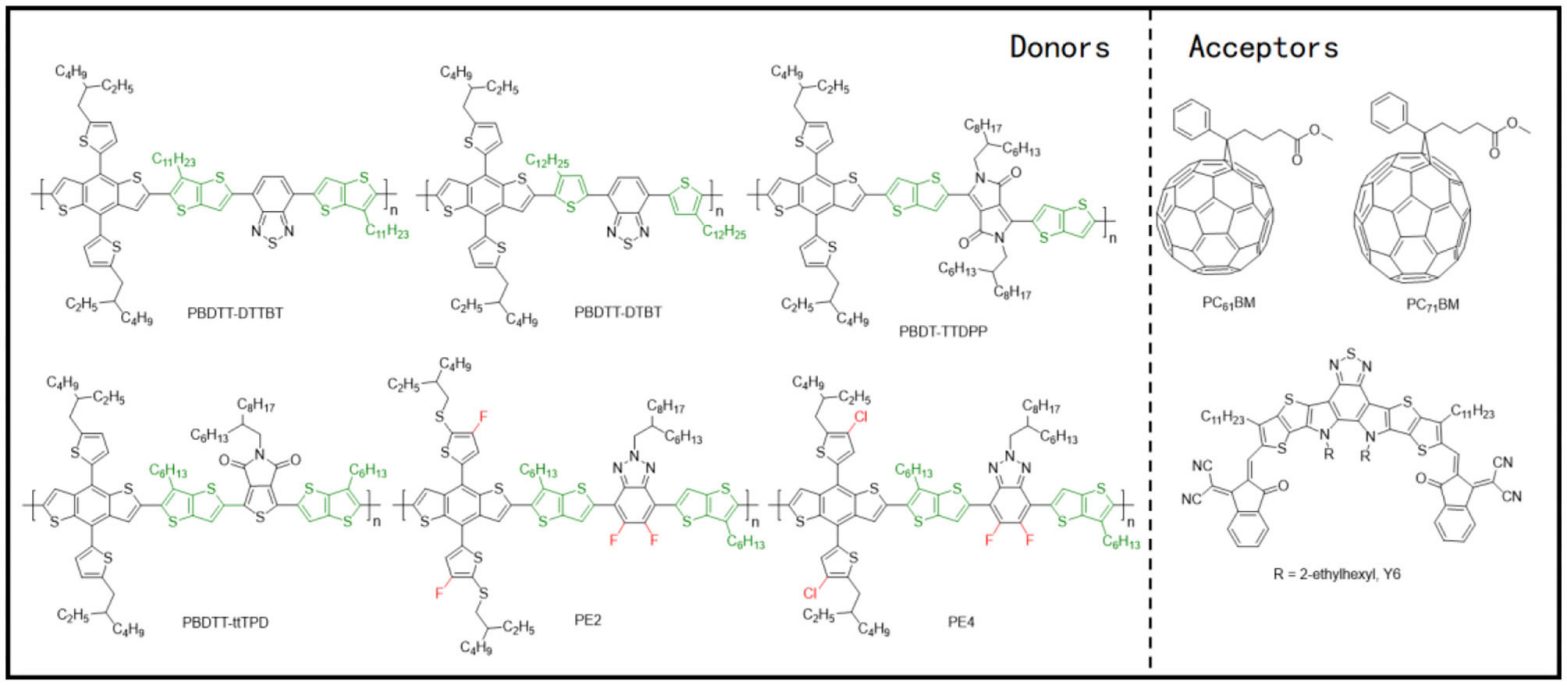
Molecular structures of representative D–π-A copolymers with TT π-bridge and mentioned acceptors.

By applying T and TT as the π-bridges, Guo et al. polymerized BDT-T and BT units and acquired two polymer donors, PBDTT–DTTBT and PBDTT–DTBT (Figure 12) (Guo et al., 2012). PCEs of 6.03 and 2.34%, respectively, have been attained for the corresponding blends with PC_71_BM, as the PBDTT–DTTBT–based system has been able to produce two orders of magnitude higher hole mobility of 1.97 × 10^−3^ cm^2^/V · s (Figure 11H), as compared to PBDTT–DTBT (1.58 × 10^−5^ cm^2^/V · s), as a consequence of good crystallinity in the former. This huge difference in mobility and device performance has been attributed to the unequal conjugated areas of the TT and T π-bridges. Likewise, the polymer based on BDT and diketopyrrolopyrrole (DPP) units have also been modified by the application of TT π-bridge, whereby forming blends with PC_71_BM as acceptor revealed a similar pattern as mentioned previously (Li et al., 2013). Moreover, an alkyl substituted TT π-bridge, 6-alkylthieno[3,2-b]thiophene has also been researched, where a PCE of 6.81% has been achieved for a PBDTT–ttTPD:PC_71_BM–based system (Figure 11I) (Kim et al., 2014).

NFAs have also been involved in the investigation of D–π-A copolymer with TT π-bridges. Chen et al. replaced the T π-bridge of J52-SF with alkyl-substituted TT π-bridge and synthesized PE2 (Figure 12). This ultimately led to down-shifted HOMO energy levels, higher crystallinity, and stronger π-π stacking in the polymer (Figure 11J), and blending it with Y6 acceptor resulted in an impressive PCE of 13.50% (Chen et al., 2019). Later, a chlorinated homolog of PE2, PE4, has been reported by the same group (Figure 12), which displayed an excellent PCE of 14% when blended with a Y6 acceptor (Figure 11K) (Tang et al., 2019).

In fact, the latter three parts can be regarded as D–π-A copolymers containing thiophene, bithiophene, and thienothiophene units. In these materials, thiophene, bithiophene, and thienothiophene units can provide a larger range for π-electron delocalization and promote molecular packing. However, there are three main differences between them. First, from the perspective of synthesis, the synthesis steps and costs of thienothiophene units are higher than the thiophene and bithiophene units. Second, from the perspective of molecular packing, the thienothiophene can provide a larger molecular conjugate plane as compared to the other two units and thereby promotes the molecular packing to the greatest extent. Finally, from the perspective of device performance, although the final film orientation and molecular packing get affected by all three units differently, and as an excellent device performance requires a proper rather than very strong crystallinity, there is no specific rule that can determine the impact of the three units on device performance, without further analyzing the specific donor and acceptor materials relationship.

The BHJ OSC devices' performance of the presented materials is summarized in Table 1.

**Table 1:** The BHJ OSCs devices performance of the presented materials.

**Active layer**	***J*_**SC**_ (mA · cm^**−2**^)**	***V*_**OC**_ (V)**	**FF(%)**	**PCE (%)**	**References**
P3HT:PC_61_BM	10.6	0.61	67.4	4.37	Li et al., 2005
P3HT:IDTBR	13.9	0.72	60	6.30	Holliday et al., 2016
P3HT:EH-IDTBR	12.1	0.76	62	6.00	Holliday et al., 2016
P3HT:IDTBR:IDFBR	14.4	0.82	64	7.7	Baran et al., 2017
P3HT:SF(DPPB)_4_	8.29	1.14	55	5.16	Li S. et al., 2016
PDCBT:PC_71_BM	11.0	0.91	72	7.2	Qin et al., 2016
PDCBT:ITIC	16.50	0.94	65.67	10.16	Qin et al., 2016
PTB1:PC_71_BM	15.0	0.56	63.3	5.6	Liang et al., 2009
PTB7:PC_71_BM	14.50	0.74	68.97	7.40	Liang et al., 2009
PTB7-Th:PC_71_BM	14.02	0.79	69.1	7.64	Liao et al., 2013
PTB7-Th:ITIC	14.21	0.81	59.1	6.80	Lin et al., 2015
PTB7-Th:IHIC	19.01	0.754	68.1	9.77	Wang et al., 2017
PTB7-Th:TPB	17.9	0.79	58	8.47	Wu et al., 2016
PTB7-Th:PNDI-T10	12.9	0.83	71	7.6	Li et al., 2016b
PBDTTT-E-T:PC_71_BM	14.59	0.58	62.6	6.21	Huo et al., 2011
PBDTTT-C-T:PC_71_BM	17.48	0.74	58.7	7.59	Huo et al., 2011
PBFTT:IT-4Cl	19.7	0.76	73.9	11.1	Su et al., 2019
PBDB-T:PC_61_BM	10.68	0.86	72.27	6.67	Qian et al., 2012
PBDB-T:ITIC	16.81	0.899	74.2	11.21	Zhao W. et al., 2016
PBDB-T:IDT-OB	16.18	0.88	71.1	10.12	Feng et al., 2017
PBDB-T:PZ1	16.05	0.830	68.99	9.19	Zhang J. et al., 2017
PM6:PC_71_BM	12.7	0.98	74	9.2	Zhang et al., 2015
PM6:SeTIC4Cl	22.92	0.78	75	13.32	Wang et al., 2018
PM6:BTP-4F	24.9	0.834	75.3	15.6	Cui et al., 2019
PM6:BTP-4Cl	25.4	0.867	75.0	16.5	Cui et al., 2019
PFBDB-T:C8-ITIC	19.6	0.94	72	13.2	Fei et al., 2018
PBDB-T-SF:IT-4F	20.50	0.88	71.9	13.0	Zhao et al., 2017
PBDB-T-2Cl:IT-4F	21.8	0.86	77	14.4	Zhang et al., 2018
J51:N2200	14.18	0.83	70.24	8.27	Gao et al., 2016a
J51:ITIC	16.47	0.82	69	9.26	Gao et al., 2016b
J52:IEICO-4F	21.9	0.734	58.5	9.3	Yao et al., 2017
J61:m-ITIC	18.31	0.912	70.77	11.77	Yang et al., 2016
J71:ITIC	17.32	0.94	69.77	11.41	Bin et al., 2016
J91:m-ITIC	18.03	0.984	65.64	11.63	Xue et al., 2017
PTzBI:ITIC	18.29	0.87	64.34	10.24	Fan et al., 2017b
PTzBI-DT:ITIC	16.84	0.91	61.53	9.43	Fan et al., 2017b
FTAZ:PC_61_BM	9.90	0.814	74.3	5.99	Lin et al., 2018
FTAZ:IDIC	21.4	0.840	67.5	12.14	Lin et al., 2018
D18:Y6	27.70	0.859	76.6	18.22	Liu et al., 2020
PDBT-T1:SdiPBI-S	11.65	0.90	65.5	7.16	Sun et al., 2015
PDBT-T1:SdiPBI-Se	12.48	0.947	69.7	8.4	Meng et al., 2016b
PDBT-T1:TPH	12.01	0.968	70.1	8.28	Meng et al., 2016a
PDBT-T1:TPH-Se	12.53	1.001	71.7	9.28	Meng et al., 2016a
PDBT-T1:ITIC-Th	16.24	0.88	67.1	9.6	Lin et al., 2016
PzNDTDTBT:PC_71_BM	10.46	0.74	65.5	5.07	Shi et al., 2013
PzNDT-T-BDD:IDIC	15.65	0.875	71.05	9.72	Jiang et al., 2018
PffBT4T-2DT:SF-PDI_2_	11.1	0.99	58	6.3	Zhao et al., 2015
PffBT4T-2DT:IDTBR	15.2	1.08	62.2	10	Baran et al., 2016
PffBT4T-2OT:PC_71_BM	17.5	0.75	72.4	9.2	Zhang et al., 2019
PFBT4T-C5Si-50%	16.98	0.75	70.98	9.26	Liu et al., 2017
PFBT4T-C5Si-25%	18.93	0.76	74.4	10.95	Liu et al., 2017
P3TEA:SF-PDI_2_	13.27	1.11	64.3	9.5	Liu J. et al., 2016
P3TEA:FTTB-PDI4	14.05	1.14	66.4	10.58	Zhang Z. G. et al., 2017
PTFB-O:ITIC	15.5	0.92	70	9.9	Li et al., 2016a
PvBDTTAZ:IDTBR	16.44	1.084	64.4	11.6	Chen et al., 2017
PBTZ-C4T:ITIC-Th	16.59	0.84	66.65	9.34	Guo et al., 2019
PTPD3T:PC_71_BM	12.3	0.786	78.7	7.72	Guo et al., 2013
PBTI3T:PC_71_BM	12.8	0.850	76.3	8.42	Guo et al., 2013
PTB7-Th:f-FBTI2-T	13.60	1.05	56.5	8.1	Sun et al., 2019
PBDTT–DTTBT:PC_71_BM	12.46	0.78	62.0	6.03	Guo et al., 2012
PBDTT–DTBT:PC_71_BM	5.78	0.82	49.5	2.34	Guo et al., 2012
PBDT-TTDPP:PC_71_BM	14.58	0.73	50	5.34	Li et al., 2013
PBDTT–ttTPD:PC_71_BM	11.05	0.84	70	6.81	Kim et al., 2014
PE2:Y6	23.24	0.83	70	13.50	Chen et al., 2019
PE4:Y6	22.21	0.84	75.43	14.02	Tang et al., 2019

## Methods of Controlling the Crystallinity of Blend Films Based on Polymer Donors

Crystallization can be considered as a process of molecular aggregation changes under the influence of postprocessing conditions. Therefore, the nature of a certain molecule and postprocessing conditions have a decisive effect on the crystallization process and the final film morphology. The modification of the crystallinity and crystallization process has also been summarized according to this idea.

### Polymer Molecular Structure

The driving force for the crystallization process is essentially the overlapping of π electron orbitals between the involved molecules and the increase of the delocalization range of π electrons. At the same time, the molecular structure determines the distribution of π electrons in the molecule. Therefore, controlling the molecular structure of the polymer can be regarded as the fundamental methodology to adjust the crystallinity and the crystallization process in the blend film. Generally, an increase in the size of the conjugated plane, side-chain engineering, and halogenation (mainly fluorination and chlorination) are the most commonly used methods for adjusting the molecular structure of polymers.

#### Increasing the Conjugated Plane

Having an enlarged conjugated plane generally means more π electrons, as well as wider delocalized π orbitals in the system, which ultimately leads to enhanced interaction between the involved crystalline organic semiconductor molecules. Therefore, a larger conjugated plane with high flatness usually is an indicator of strong crystallinity. The most representative instances in this regard are the polymers with a TT π-bridge, as mentioned previously (Guo et al., 2012; Li et al., 2013; Kim et al., 2014; Chen et al., 2019; Tang et al., 2019). Similarly, polymers based on BDT-T or DTBDT units usually exhibit improved crystallinity than the BDT series. As illustrated in Figure 6C, the BDT-T–based D-A copolymer, PTB7-Th, showed an enhanced signal peak in the GIWAXS spectrogram as compared to its corresponding BDT-based homolog, PTB7, indicating that increased conjugation plane can indeed enhance the intermolecular aggregation effect.

#### Side-Chain Engineering

Side chains in organic semiconductor materials are generally introduced for solubilization, but they also affect the interaction between the molecules as well. Alkoxycarbonyl, alkyl, alkoxy, thioether groups, and silyl groups have often been reported as the most used side chains (Fan et al., 2017a, 2018; Liu et al., 2018). The interaction between the side chains and the resulting changes in the molecular configuration generally affects the molecules' self-aggregation effect and thus affects the crystallinity. Unfortunately, there is no obvious rule to indicate which side chains can increase or decrease the molecular crystallinity. On the other hand, the structure of the side chains itself, as well as the connecting positions, also influences the crystallinity of molecules. Shi et al. synthesized a series of PffBZ copolymers with different side chains and revealed that the performance of the polymers with branching on the second position of the alkyl chain and the third position of the alkoxy chain delivered the best film morphology and molecular packing characteristics (Shi et al., 2020). Furthermore, adjusting the crystallinity through the molecular structure will also affect the energy levels, absorption, and other properties of the material. The systematic study of different side chains in different systems is an important research direction.

#### Halogenation Strategy

The halogenation strategy has been proven to be one of the simplest and most effective methods to improve the performance of both polymer donor– and NFA-based systems. Extensive research on fluorination methods has shown that the introduction of the fluorine atoms, having the largest electronegativity and small atomic radius, into organic semiconductor materials can significantly decrease the energy levels and improve molecular aggregation (Zhang et al., 2016; Xue et al., 2017). In this regard, by fluorinating the BT unit of PBnDT-DTBT, a D–π-A polymer, the researchers obtained PBnDT-DTffBT polymer that displayed increased aggregation as its thin films revealed an additional absorption shoulder peak in the spectra (Zhou et al., 2011). Similarly, a BDD-bithiophene–based polymer, PBDD4T-2F, with two fluorine atoms connected to two adjacent thiophenes showed stronger crystallinity than its non-fluorinated homologs, which led to relatively more complex absorption curves, as well as stronger peaks in the GIWAXS spectra (Zhang et al., 2016). Likewise, compared with the fluorine atoms, the chlorine atoms not only have a larger radius but are also capable to lower the corresponding energy levels even further, as their 3d orbitals can accept electrons from the conjugated skeleton. Furthermore, the chlorinated polymers are relatively easier to synthesize than the fluorinated polymers. Mo et al., in this regard synthesized PBDTHD-ClBTDD, a D–π-A copolymer based on BDT-T and BT units with a chlorine atom attached to the BT unit, whose absorption spectra revealed a stronger vibrionic shoulder, suggesting stronger π-π stacking in the system (Mo et al., 2017).

### Post-processing Conditions

Solution processing is one of the major advantages that organic semiconductors have over their inorganic counterparts. During the spin coating process, the active layer solvent gradually volatilizes, and as a result, the solute molecules approach each other, and eventually, a film is formed. Because the fast cooling process is involved here, the post–film formation processes have a strong influence on the crystallization process and film morphology of the corresponding films. The utilization of solvent additives and thermal annealing can be regarded as two important external parameters that can significantly impact the final film characteristics.

#### Solvent Additives

Trichloromethane or chloroform (CF) is the most frequently used solvent for film preparation because of its excellent dissolution ability and low boiling point. However, the latter also results in the rapid drying of the films, leading to poor and unstable film morphologies (Li et al., 2013). To overcome this, a high boiling point additive has been introduced in the system that can lower the drying process and hence allow sufficient time to the corresponding active layer components for proper self-organization. As a result, an enhanced film morphology and ultimately device performance will be achieved. In this regard, DIO can be termed as the most commonly used additive to improve the performance of devices based on polymer materials such as PM6, PTB7-Th, PBDB-T, etc. (Zhang et al., 2015; Liu F. et al., 2016; Zhao W. et al., 2016).

#### Thermal Annealing

Thermal annealing can be regarded as a simple, widely used, and cost-efficient strategy that can effectively modify the crystallinity of the blend films in a controllable fashion. At high temperatures, the molecules in the films can self-assemble to repair the defects induced by the spin-coating crystallization process and ultimately improve the morphology of the films. Researches have shown that thermal annealing is effective for improving the exciton diffusion for some organic donor materials (Long et al., 2017). For PffBT4T-2OD polymer, composed of BTz and bithiophene units, Zhang et al. investigated the influence of thermal annealing on crystallite size, exciton diffusion, and charge harvesting in films and stated that thermal annealing increases domain size and exciton diffusion length simultaneously in the corresponding system (Zhang et al., 2019).

In contrast to the methods for regulating the molecular structure, the external parameters of film preparation enable better control of the molecular crystallization process and the film morphology with almost no other influence. Coupled with the simple operation, this methodology is more suitable for the improvement of film morphology and photovoltaic performance. Precise and utmost control, however, remains a complicated and difficult task.

### Compatibility Between Donor and Acceptor

Even though there are at least two substances in the blend films forming the BHJ, the adjustment methods mentioned previously are largely aimed at a single substance, either donor or acceptor. Hence, things get quite complex when we talk about controlling the BHJ parameters. Compared with a neat film of a certain substance, compatibility between donor and acceptor can be regarded as the most essential concept in OSC active layer. Compatibility generally refers to the ability of two molecules to mix; however, the extent of it should neither be too excessive nor inadequate, just like crystallinity, as extensive compatibility will result in poor phase separation, small domain size, and low phase purity, whereas weak compatibility will result in excessively large grains. Furthermore, the compatibility between the two molecules is closely related to their respective structures and their interactions. It has been reported that many factors such as similar chemical structure, surface energy, molecular orientation, and crystallite and domain size will definitely affect the compatibility (Mai et al., 2016). For instance, Li et al. synthesized two difluorinated copolymers based on BTz and bithiophene units, PTFB-O and PTFB-P (Li et al., 2016a). The only difference between them is the position where the two fluorine atoms are substituted. Thus, this small detail led to different stable molecular configurations, different crystallinity, and different compatibility with the PC_71_BM and ITIC acceptors. As a result, while the PTFB-O yielded >10% and 6.5% PCE for NFA and fullerene-based cells, respectively, the PTFB-P was found to be a much better match for the fullerene acceptor.

Compatibility is a relatively vague concept as it is almost impossible to predict whether the compatibility between two molecules is appropriate and can only be inferred from the results of relevant film characterization. Hence, the adjustment of the compatibility between two molecules remains a research gap in the BHJ OSC field.

## Summary and Outlooks

In the past few decades, the performance of BHJ OSCs has experienced rapid progression, largely due to the continuous optimization and development of polymer donor and NFA materials. In this short review, structures, crystallization, and packing characteristics of representative polymer donors with various acceptors have been summarized, followed by methods for controlling the crystallinity and crystallization process of the relevant moieties. Thanks to the continuous efforts by outstanding scholars for the BHJ OSCs, this field is now seen as having great potential for industrial applications. However, numerous issues still need to be solved, which prevents further breakthroughs in the device performance, especially PCE.

In the past few years, most of the molecular design and development have been based on a trial model, without a great deal of theoretical guidance, as the theoretical research in the BHJ OSC field is not yet mature enough. For example, although it is a generally accepted consensus that uniform nanoscale interpenetrating network morphology and face-on–oriented molecular orientation are crucial to favorable FF, how to accurately control the material properties and device preparation conditions to obtain satisfactory FF remains an urgent problem. The development of theories that can directly correlate the concepts of polymer molecular structure, crystallinity, morphology of films, and FF of OSCs may provide new ideas for molecular design.

Second, compared with laboratory research, large-area devices are one of the most significant requirements for industrial applications. The preparation process of large-area OSCs often means a thicker and uneven active layer, which further poses more difficult challenges for the OSC materials. Compared with the small molecules, the highly crystalline polymer donors enable the corresponding films to show acceptable morphology, and thus, the resulting OSC devices can maintain a certain excellent performance, which still, however, is not up to the laboratory-scale standards. Hence, by taking advantage of the strong crystallinity of certain polymer donors, optimal self-assembly in the thick film preparation can be realized, and ultimately, thick film system OSCs with as little efficiency loss as possible can be obtained, thus solving the current issue in hand.

Finally, the environmental pollution during the preparation of OSCs, and the polymer donor structure-dependent instability of OSCs are also important problems that need to be resolved. The large conjugated planes of the polymer donor materials, even though enduing them with a strong self-aggregation effect and crystallinity, also make them less soluble. As a result, almost all OSCs based on polymer donor materials are processed using toxic CF or chlorobenzene solvents, and common environmentally friendly solvents such as water and alcohol are rendered completely unusable. Furthermore, the polymer donors generally contain abundant π electrons and hence are prone to redox reactions when exposed to air and light, which brings severe device stability problems. How to increase the solubility and stability of the common polymer donor materials while maintaining strong crystallinity requires some more thinking to do.

In short, great progress has been made in the research of OSCs based on polymer donor materials, which makes the use of solar energy more convenient and a big step forward in bringing new vitality to the photovoltaic field. If the current progress can go further in terms of energy conversion efficiency and stability, this technology will be widely used in daily life, with exceptional market prospects. We believe that having some outstanding scientific researchers in the field who are tirelessly working on material design and synthesis, theoretical research and development, and device preparation and optimization, the BHJ OSCs will gradually mature in the next few years and become an indispensable part of the entire social energy structure.

## Author Contributions

DQ and KL prepared the manuscript. MA and ZW helped to prepare and revise the manuscript. KL and ZW supervised the project. All authors discussed and commented on the paper.

## Conflict of Interest

The authors declare that the research was conducted in the absence of any commercial or financial relationships that could be construed as a potential conflict of interest.
